# Methylation of Cytidine 1407 Increases the Lifetimes of the A‑Site Ground and Excited States of *E. coli* 16S Ribosomal RNA

**DOI:** 10.1021/jacs.5c06523

**Published:** 2025-07-17

**Authors:** Stefan Hilber, Alessandro Marotto, Christoph Mitteregger, Martin Tollinger, Christoph Kreutz

**Affiliations:** † Institute of Organic Chemistry and Center for Molecular Biosciences Innsbruck (CMBI), 27255 University of Innsbruck, Innrain 80/82, 6020 Innsbruck, Austria

## Abstract

The C5 methylation of cytidine 1407 (C1407) in the *E. coli* 16S rRNA is a permanent modification. The methylation occurs directly at the A-site internal loop, a known dynamic hotspot in helix 44 (H44) that undergoes conformational exchange between a functional ground and an excited state. Here, we show by relaxation dispersion NMR spectroscopy that methylation of C1407 reshapes the folding kinetics of the A-site RNA and prolongs the lifetimes of its ground and excited states. Carr–Purcell–Meiboom–Gill relaxation dispersion experiments of unmodified and m^5^C1407-modified A-site RNA reveal that C5 methylation slows down the transition rate between the ground and excited states by 1.6-fold. This kinetic effect increases the lifetimes of the excited and ground states, with the latter playing a critical role in the correct codon anti-codon recognition during the elongation phase of translation. Thus, C5 methylation of C1407 functions as a kinetic regulator in the *E. coli* rRNA to properly adjust the time frame for the selection of the correct tRNA–mRNA interaction.

## Graphical abstract

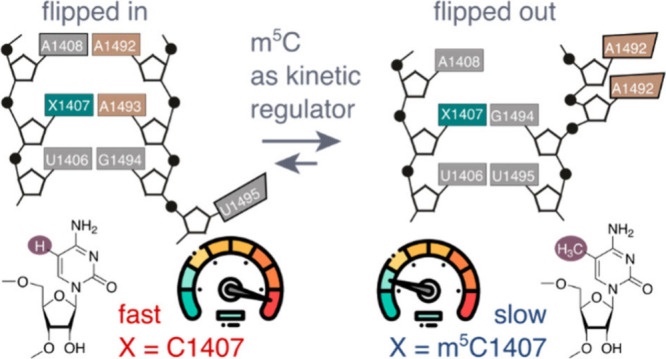

The positions and types of modified nucleotides in various RNA species are well characterized; however, numerous others remain unidentified, undetected, or transient in nature and are therefore not yet fully described.^1^ One of the most common modifications is methylation. For example, methylation of the exocyclic amino group at position 6 of adenosine, giving *N* ^6^-methyladenosine (m^6^A), has an important role in RNA metabolism and a growing number of cellular processes.^2^ One role of m^6^A is the recruitment of reader proteins specifically recognizing the methyl group, but it was recently shown that this methylation can also influence the hybridization kinetics in double-stranded RNA, suggesting an active role of methylations in reshaping the folding landscapes of RNA.^3^ Methylations are also among the most common modifications in bacterial rRNAs, with 24 methylation sites reported in mature *E. coli* ribosomes.^4^ Among these, three 5-methylcytidines (m^5^Cs) are known, located at positions 967 and 1407 in the 16S rRNA and at position 1962 in the 23S rRNA (Figure 1A). The methyl group at C1407 is transferred by protein *YebU* and is a permanent modification.^5^ The nucleotide C1407 is directly located at a functionally important region, the so-called ribosomal A-site internal loop, which plays an essential role in decoding the mRNA. The correct codon–anti-codon mini-helix formed between the cognate aminoacyl tRNA and mRNA is stabilized by flipping out two internal-loop adenines (A1492 and A1493), which are located close to C1407 (Figure 1B).^6^ It was earlier shown that this region undergoes an exchange process on the micro- to millisecond time scale, involving an excited state in which three noncanonical base pairs (A1492•A1408, A1493•C1407, and G1494•U1406) are formed.^7^ Here, we report a Carr–Purcell–Meiboom–Gill (CPMG) relaxation dispersion (RD) NMR study, which shows how methylation of C1407 changes the kinetics of this refolding process and takes an active role in the kinetic reshaping of the A-site RNA’s folding landscape. The 5-methyl of C1407 slows the exchange process between the ground state (GS) and the excited state (ES), while the populations of the two states remain virtually unchanged. In m^5^C1407-modified RNA, the increased activation energy barrier between the ground and excited state thus enhances the lifetimes of the individual states, with the m^5^C modification acting as a kinetic regulator to properly adjust the time frame for the selection of the correct tRNA–mRNA interaction.

**Figure 1:**
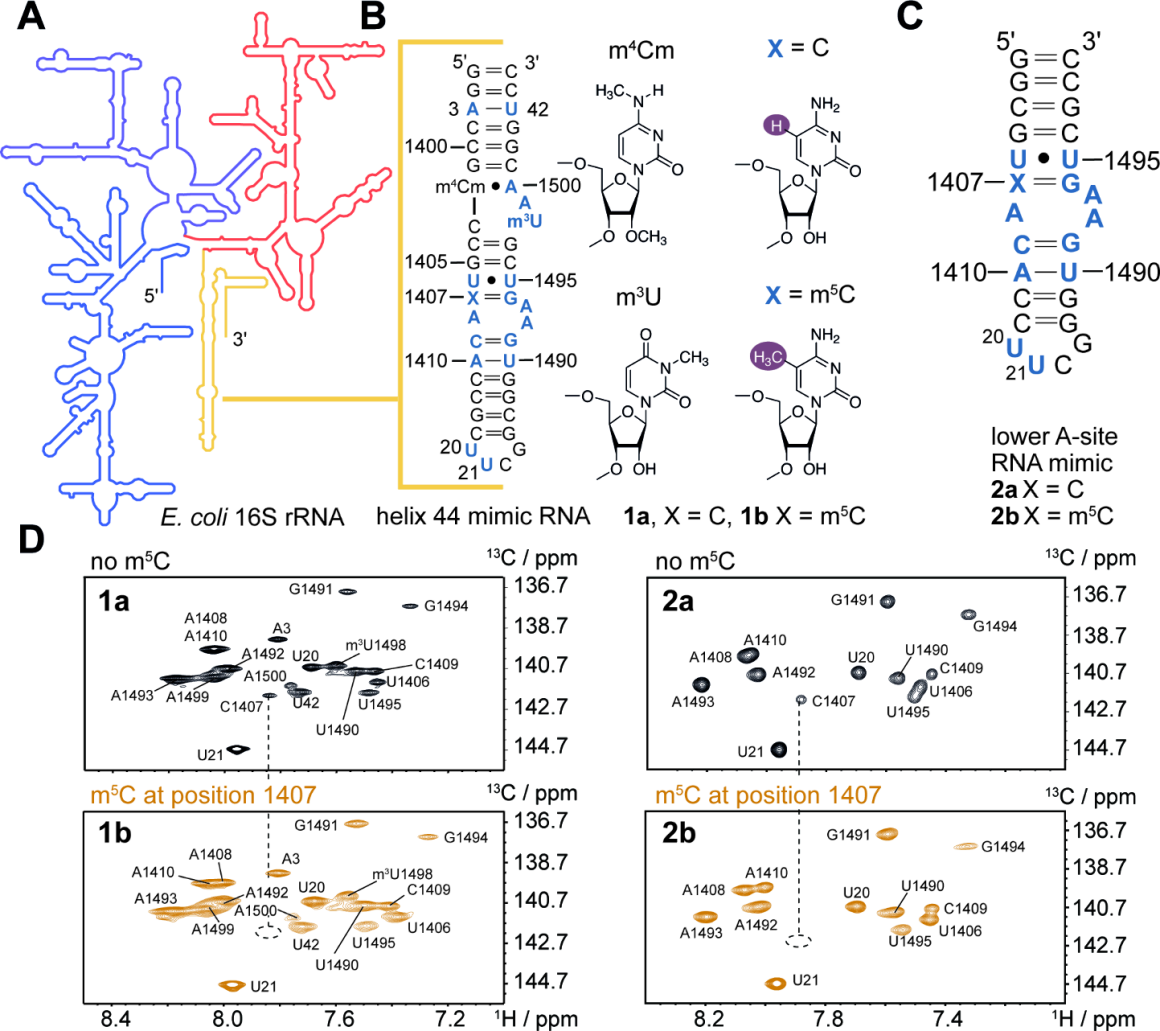
H44 mimics used for the relaxation dispersion NMR study and the respective CH correlation spectra. **A)** Schematic representation of the *E. coli* 16S rRNA with the H44 sequence part highlighted in yellow. **B)** 44 nt H44 mimics **1a** and **1b** without and with the m^5^C1407 modification. The ^13^C6 pyrimidine/^13^C8 purine labeled nucleotides are highlighted in blue. **C)** 27 nt H44 mimics **2a** and **2b** without and with m^5^C1407 modification. The ^13^C6 pyrimidine/^13^C8 purine labeled nucleotides are highlighted in blue. **D)** ^1^H–^13^C-HSQC spectra of all four constructs.

We used RNA solid phase synthesis to produce a 44-nucleotide (nt) H44 mimic containing the naturally occurring modified residues 2′-*O*-methyl-*N* ^4^-methylcytidine (m^4^Cm) and N^3^-methyluridine (m^3^U). Two constructs were produced: **1a** without methylation of C1407 and **1b** with the m^5^C1407 modifier (Figure 1B). Furthermore, the smaller 27 nt constructs **2a** and **2b** were synthesized to investigate the effect of the m^5^C1407 modification on the earlier reported exchange kinetics of the A-site RNA (Figure 1B and C). All four constructs included ^13^C6/^2^H5 pyrimidine and ^13^C8 purine labeled nucleotides to allow for the application of relaxation dispersion experiments. Site-specific ^13^C labeling made the acquisition of ^1^H–^13^C-HSQC spectra of the 44 and 27 nt A-site RNAs with and without m^5^C possible; all resonances could be unambiguously assigned (Figure 1D). For the 44 nt RNA a previously reported combinatorial approach capitalizing on solid phase RNA synthesis and residue-specific stable isotope labeling was applied.^8^ The proton–carbon correlation peaks for residues 1406 to 1410 and 1490 to 1495 showed chemical shift values very similar to those reported by Al-Hashimi and co-workers,^7^ confirming that the GS structure with flipped out A1492 and A1493 is preserved in the larger H44 mimics **1a** and **1b**, both with and without m^5^C1407, as in the smaller constructs **2a** and **2b**.

We then investigated the dynamic behavior of the four RNAs via ^1^H and ^13^C CPMG RD NMR experiments. Nonflat dispersion profiles were observed for residues in all four RNAs, indicating dynamics on the millisecond time scale (Supporting Figures 3, 4, 5, and 6). For the 44 nt RNAs **1a** and **1b**, these residues included the A-site loop (1406–1410 and 1490–1495), as well as residues in the m^3^U bulge (m^3^U1498 and A1499). Millisecond dynamics in the m^3^U bulge have not been reported previously. Statistical tests based on the Akaike information criteria (AICc), assuming two exchange processes (Supporting Table 2), indicated that the time scales of the processes in the A-site and the m^3^U bulge are not identical (exchange rate constant *k*_ex_ > 8000 s^–1^ for the m^3^U bulge, and *k*_ex_ < 6000 s^–1^ for the A-site, Supporting Table 3).

In addition, the data indicated that methylation at C1407 slowed down *k*_ex_ in the A-site of RNA **1b** to ≈4000 s^–1^. To further investigate this effect in the A-site, we resorted to the lower molecular weight constructs **2a** and **2b**, which exhibited improved relaxation behavior, i.e., lower *R*_2,eff_ rates and less data scattering in the dispersion profiles. Figures 2C and 2D display the ^13^C and ^1^H RD profiles of U1495 near the methylation site C1407. For both RNAs **2a** and **2b**, the experimental ^1^H and ^13^C dispersion profiles could be fitted to global exchange processes, assuming a common set of kinetic parameters for the A-site, along with residue-specific chemical shift differences, as verified by AICc statistical tests (Supporting Tables 4, 5, and 6).

**Figure 2:**
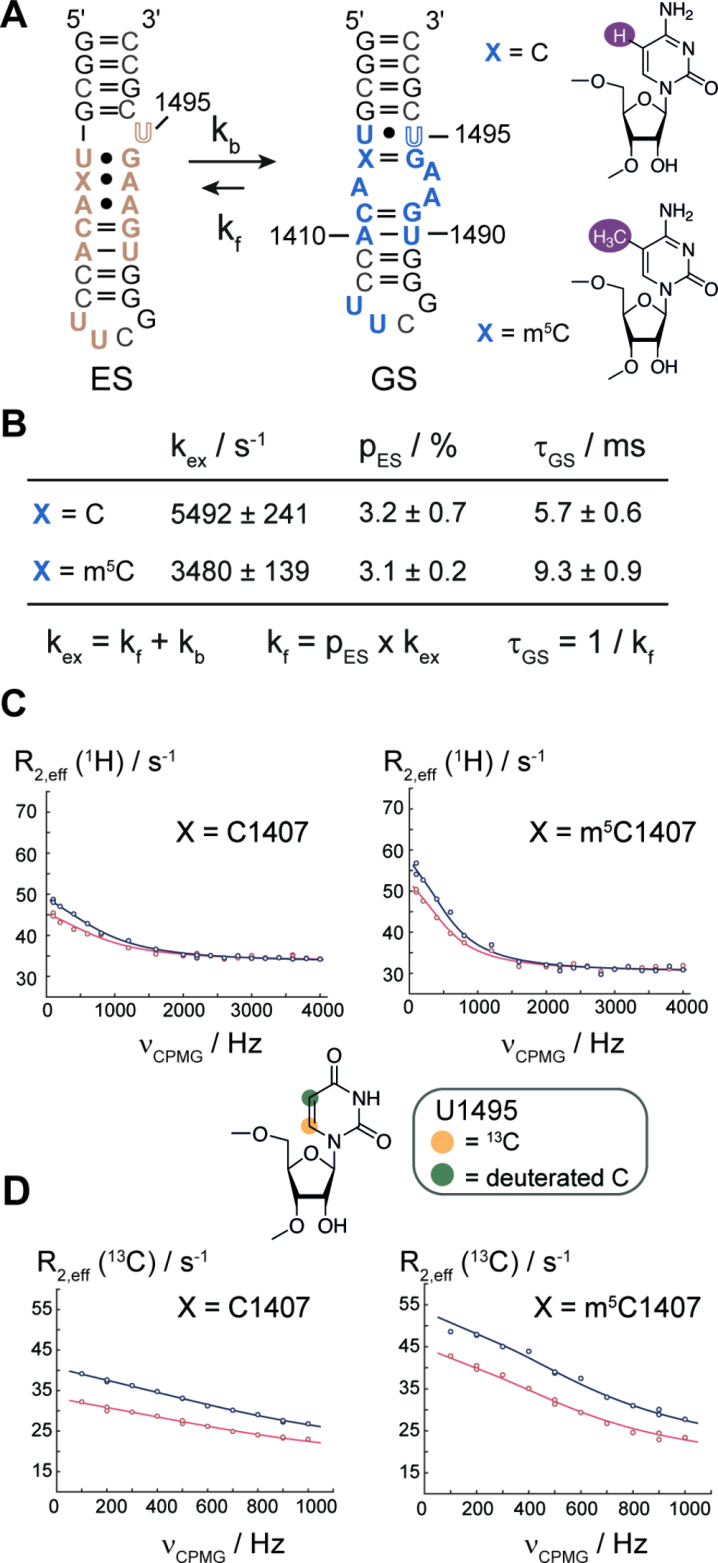
RD NMR data of C1407- and m^5^C1407-modified RNAs **2a** and **2b**. **A)** Secondary structure representation of the A-site RNA exchange process between the GS and the ES. The ^13^C6 pyrimidine/^13^C8 purine labeled nucleotides are highlighted ( blue GS, pale taupe ES). **B)** Global fitting results for RNAs **2a** and **2b**. **C)** ^1^H RD profiles and **D)** ^13^C RD profiles of U1495 in the unmodified and m^5^C1407 A-site RNA.

The chemical shift difference signatures strongly suggest that in RNAs **2a** and **2b** the same exchange process between A1493/1493 flipped-in (ES) and A1493/1493 flipped-out (GS) is present as reported by Al-Hashimi and co-workers (Figure 2A).^7^ While the excited and ground state populations of the two RNAs **2a** and **2b** are identical and in perfect accordance with the original data of the unmodified RNA (∼3% Al-Hashimi and co-workers,^7^ **2a** 3.2 ± 0.7% and **2b** 3.1 ± 0.2% in our current work), the transition rate between the two states is slower in the methylated form, **2b**, by a factor of 1.6 compared to the unmethylated form, **2a** (Figure 2B), confirming (within error) the observations made for RNAs **1a** and **1b**. This suggests either a higher-energy transition state and/or that both the ground and excited states are stabilized by methylation of C1407. Support for the latter model comes from the reported stabilizing effect of a pyrimidine 5-methyl group in duplex structures.^9^ To test this, we recorded UV melting curves of RNAs **2a** and **2b** along with excited state mimics containing C1407•A1493 and m^5^C1407•A1493 base pairs (Supporting Figure 8). The data revealed a slightly stabilizing contribution of the C1407 methylation (ΔΔ*G* ^2a,2b^ = −0.5 ± 0.1 kcal mol^–1^ and ΔΔG^ES mimic^_no m5C‑m5C_ = −3.0 ± 0.6 kcal mol^–1^), suggesting that the higher activation barrier and the slower exchange rate at least in part result from thermodynamically more stable ground and excited states in the m^5^C-modified RNAs (Supporting Figure 9).

C5 methylation of C1407 slows the transition rate between the ground and excited states and increases their lifetimes in the A-site RNA. It could thus serve as a kinetic regulator to tune the kinetics of the tightly orchestrated translation elongation process (Figure 3A). Rodnina and co-workers proposed a kinetic mechanism for high-fidelity tRNA discrimination on the ribosome (Figure 3A).^10^ In *E. coli*, the translation process is very fast with elongation rates up to 20 amino acids per second, allowing ∼50 ms for the addition of a single amino acid in the multistep procedure. At this high rate, the *E. coli* cell needs to balance the speed and fidelity of amino acid addition. Conformational plasticity is intrinsically required for the elongation process, and the A-site RNA needs to switch between A1492/A1493 flipped-in and flipped-out states during P-site tRNA binding and initial A-site tRNA binding (Figure 3B).^6^

**Figure 3:**
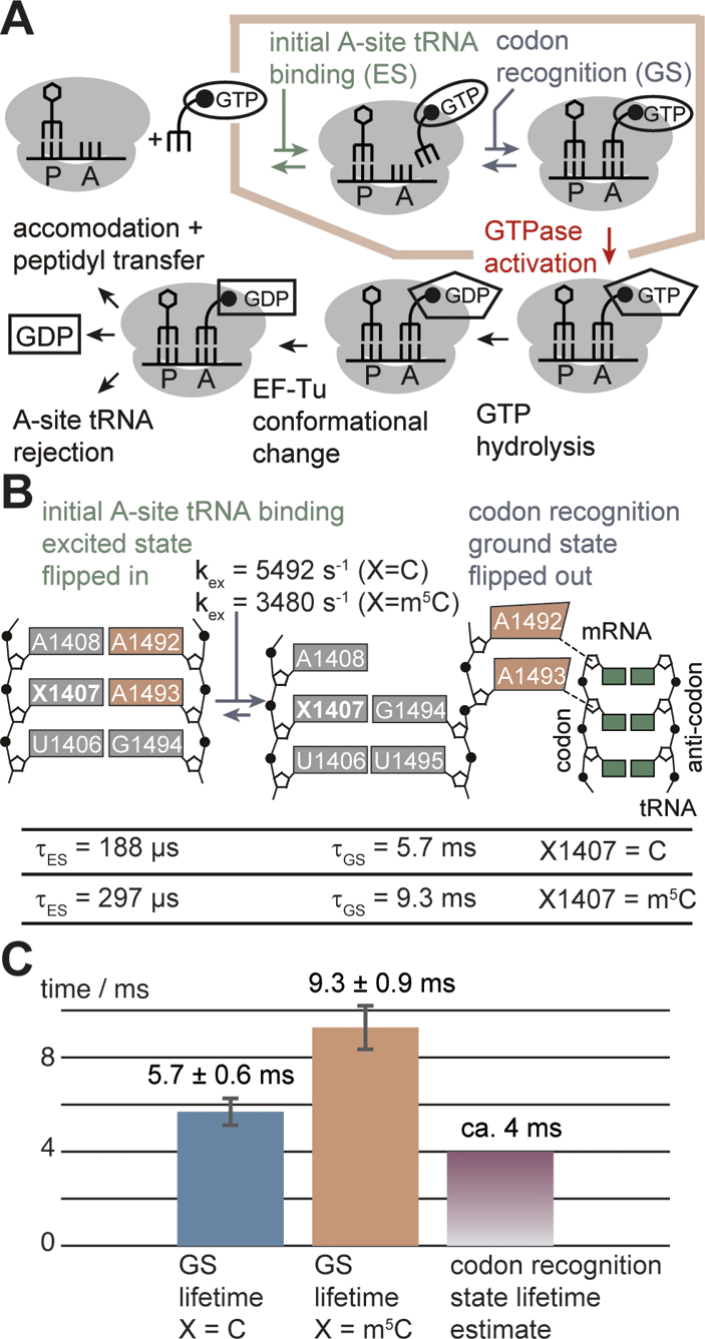
Implications of slowed exchange dynamics of the A-site RNA by the C5 methylation of C1407. **A)** Kinetic mechanism of elongation factor (EF)-Tu-dependent aa-tRNA binding to the A-site.^5^ **B)** Schematic representation of ES and GS structure of the A-site RNA and their role in the tRNA binding process to the A-site. **C)** Ground state lifetimes of unmodified and m^5^C1407-modified A-site RNA compared to the codon recognition state lifetime estimate from Gromadski et al.^10^

Initial tRNA binding is followed by codon recognition, a checkpoint for distinguishing cognate and near cognate tRNAs. We speculate that the conformational exchange process described here contributes to the kinetic tuning of these two steps. First, initial A-site tRNA binding is enabled by the A1492/A193 flipped-in ES, which is then followed by the codon recognition in the flipped-out GS-conformation.^11^ C5 methylation of C1407 kinetically modulates the transition between these two states. We propose that the methylation functions as a kinetic regulator in the H44. In the ES-conformation, with A1492/A1493 flipped-in, the initial A-site tRNA binding occurs.^11^ Then, a return to the GS-conformation is required for proofreading of the codon/anti-codon helix.^11^ C5 methylation increases the lifetimes of both the GS- and ES-conformations by a factor of 1.6. Specifically, the enhanced ES may provide the correct temporal window for the initial binding of tRNA to the A-site. The extended GS lifetime is very likely necessary for fail-safe proofreading. While the ground state lifetime of RNA **2a**, lacking the m^5^C modification (5.7 ± 0.6 ms), approaches the codon recognition state lifetime (∼4 ms) reported by Rodnina and co-workers,^10^ it is significantly extended (9.3 ± 0.9 ms) in the m^5^C-modified RNA **2b** (Figure 3C). The longer lifetime of this conformation via m^5^C1407 modification reduces the likelihood of selecting incorrect non/near-cognate aa-tRNA in this step. The extended lifetime could act as a temporal buffer for the elongation process, giving a reliably long window for codon recognition (Figure 3C).

Our results support the notion that chemical modifications fulfill important roles in shaping the conformational landscape of biologically relevant RNAs. The results add another aspect to how methylated nucleotides can adapt the kinetics of the nucleic acid folding landscape for a specific task.^3,12^ In contrast to the m^6^A modification, which slows duplex annealing by destabilization, the m^5^C modifier acts by slowing the exchange kinetics via a stabilizing effect. This illustrates that modifiers can not only directly influence RNA structure, for example, by restricting base pairing as seen with m^1^G and m^1^A, but also modulate the kinetics of an RNA’s inherent exchange processes. The stabilizing effect of m^5^C on the A-site dynamics is reminiscent of the influence of the 2′-*O*-methyl modification (N_m_), which reshuffles excited/ground state populations and increases their lifetimes.^13^ In a very recent example, Zhang and co-workers showed that in addition to the 3D ground state structure the lifetime of the lowest free energy state influences biological activity. For the Zika virus exoribonuclease-resistant RNA (xrRNA) a very long-lived ground conformational state is key to exoribonuclease resistance. Mutations that reduce its apparent lifetime without affecting the 3D structure also led to decreased exoribonuclease resistance *in vitro* and impaired virus replication in cells.^14^ Our results underscore the biological importance of the lifetime of functional ground states and the role of conformational dynamics in RNA function. They further suggest that studies of RNA modifications and their influence on function should consider both structural and kinetic effects to provide a comprehensive picture of how RNA modifications can influence biological function.

## Supplementary Material



## Notes

Details on synthetic procedures, RNA synthesis, UV melting curve experiments, NMR spectroscopy and mass spectrometry; ^1^H and ^13^C relaxation dispersion profiles of **1a**, **1b**, **2a**, and **2b**; assignment of imino resonances of **2b**; melting curve data of wt A-site and ES mimics; influence of m^5^C on the A-site RNA folding landscape (PDF)
