# Supplementary material for: Methylation of Cytidine 1407 Increases the Lifetimes of the A‑Site Ground and Excited States of E. coli 16S Ribosomal RNA
Source: J Am Chem Soc. 2025 Jul 17;147(30):26097–101. doi: 10.1021/jacs.5c06523 (PMC12314911; doi:10.1021/jacs.5c06523)
Supplement: Supplementary file 1 [file ja5c06523_si_001.pdf]

SUPPLEMENTARY INFORMATION

Methylation of Cytidine 1407 Increases the Lifetimes of the A-Site Ground and Excited States of *E. coli* 16S Ribosomal RNA

Stefan Hilber[1], Alessandro Marotto[1], Christoph Mitteregger[1], Martin Tollinger[1]*, Christoph Kreutz[1]*

[1] Institute of Organic Chemistry and Center for Molecular Biosciences Innsbruck (CMBI), University of Innsbruck, Innrain 80/82, 6020 Innsbruck, Austria.

* To whom correspondence should be addressed - email: martin.tollinger@uibk.ac.at or christoph.kreutz@uibk.ac.at

Materials and Methods

Synthesis of the (5-$^{13}CH_3$)-6-D-5-methylcytidine phosphoramidite 11

General information. NMR spectra were acquired on a Bruker Avance Neo 400 MHz instrument. Chemical Shifts are reported relative to TMS and referenced to residual proton solvent signal: $CDCl_3$ (7.26 ppm) for ^1H NMR spectra and $CDCl_3$ (77.0 ppm) for ^{13}C spectra. DMSO-d_6 (2.50 ppm) for ^1H NMR spectra and DMSO-d_6 (39.52 ppm) for ^{13}C spectra. ^{31}P shifts are reported relative to external phosphoric acid (85%). ^1H assignments are based on double quantum filtered gradient selected COSY experiments. ^{13}C shifts were assigned from gradient selected phase sensitive HSQC and magnitude HMBC experiments. Silica 60F-254 plates were used for TLC (thin layer chromatography). For FCC (flash column chromatography) silica gel 60 (230-400 mesh) was used. Reagents and solvents were purchased from Merck/Sigma-Aldrich and used without further purification. Organic solvents were extensively dried using freshly activated molecular sieves (4 Å). ^2H and ^{13}C labeled compounds were obtaiend for Cambridge Isotope Laboratories through the CIL award 2024.

Synthetic route

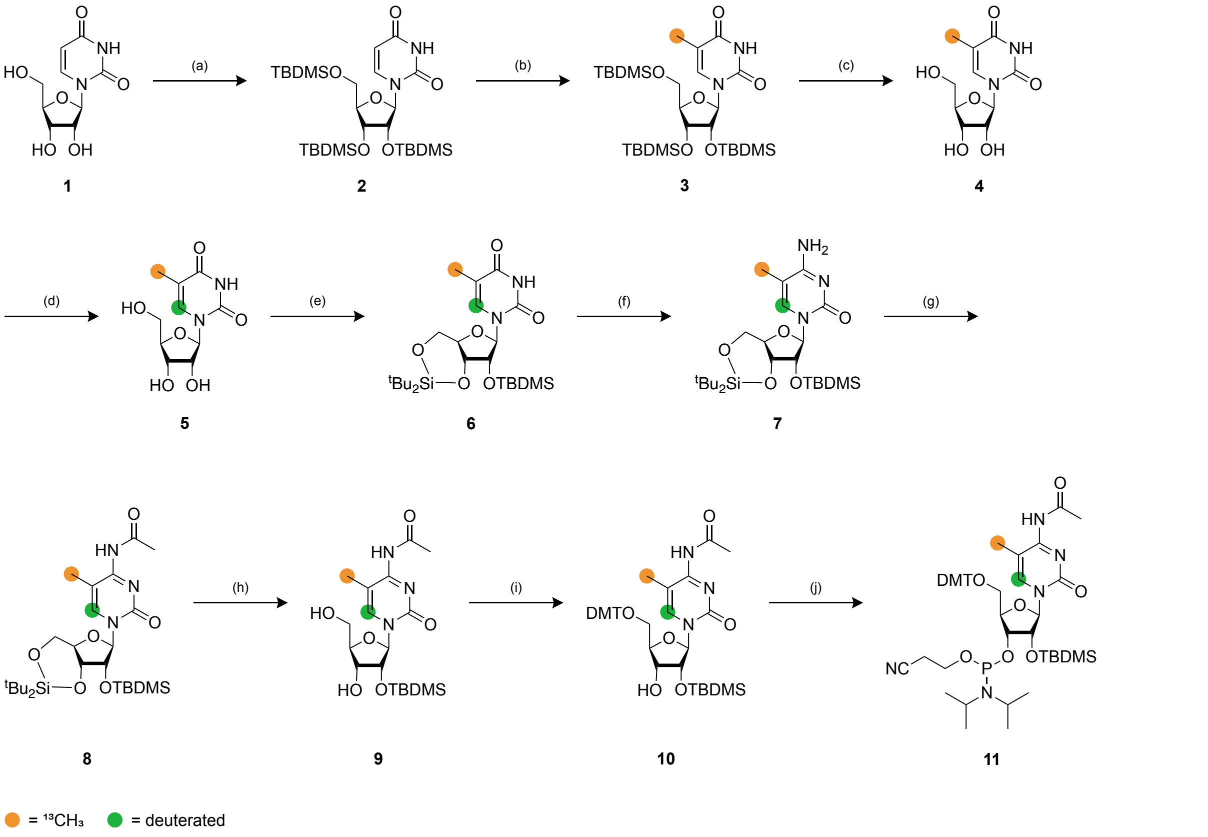

Supporting Scheme 1. Preparation of the (5-$^{13}CH_3$)-6-D-5-methylcytidine phosphoramidite building block **11**. **(a)** TBDMS-Cl, imidazole, in DMF, overnight, rt, 100%; **(b)** I. TMEDA, *sec*-BuLi in THF, 40 min, -80°C; $^{13}CH_3I$, 30 min, -80°C, 75%; **(c)** TEA·3HF in THF, overnight, rt, 100%; **(d)** D_2O, NaOH, in DMSO-d_6, 7 days, 135°C, 80%; **(e)** I. $tBu_2Si(OTf)_2$, 1h, 0°C; II. Imidazole, 30 min, rt; III. TBMDS-Cl in DMF, 2h, 60°C, 72%; **(f)** I. TIPS-Cl, NEt_3, DMAP, 2h, rt; II. $NH_{3\,aq.}$ 28% in acetonitrile, overnight, rt, 79%; **(g)** Ac_2O, DMAP in DMF, overnight, rt, 70%; **(h)** HF/pyridine, in DCM, 3h, 0°C, not purified; **(i)** DMT-Cl in pyridine, overnight, rt, 79%; **(j)** CEP-Cl, DIPEA, in THF, overnight, rt, 77%.

2′,3′,5′-Tri-*O*-(*tert*-butyldimethylsilyl)-uridine (**2**)

To a solution of uridine (**1**) (5.00 g, 20.5 mmol, 1.00 eq.) in 135 mL anhydrous DMF imidazole (9.76 g, 143 mmol, 7.00 eq.) and *tert*-butyldimethylsilyl chloride (15.4 g, 102 mmol, 5.00 eq.) were added and the solution was stirred over night at room temperature under argon atmosphere. TLC (*n*-Hex/EA: 8/2) confirmed complete conversion of the reaction, the solvent was evaporated, and the oily residue was dissolved in ethyl acetate. The solution was washed four times with saturated sodium chloride solution and once with saturated sodium bicarbonate solution. The organic layer was dried over sodium sulfate. The filtrate was evaporated to dryness and the residue was dried in *vacuo* to obtain pure compound **2** as a white foam.

Yield: 12.0 g of a white foam (20.5 mmol, 100%);

TLC: (*n*-Hex/EA: 8/2); $R_f = 0.25$;

^{1}H-NMR (400 MHz, DMSO-d_6, 25°C): δ 11.39 (d, 1H, N(3)*H*, $^4J_{HH}$=1.80 Hz); 7.77 (d, 1H, C(6)*H*, $^3J_{HH}$=8.09 Hz); 5.81 (d, 1H, C(1')*H*, $^3J_{HH}$=5.70 Hz); 5.62 (dxd, 1H, C(5)*H*, $^3J_{HH}$=8.08 Hz, $^4J_{HH}$=1.92 Hz); 4.22 (dxd, 1H, C(2')*H*, $^3J_{HH}$=5.99 Hz, $^3J_{HH}$=5.40 Hz); 4.06 (dxd, 1H, C(3')*H*, $^3J_{HH}$=4.60 Hz, $^3J_{HH}$=4.04 Hz); 3.94 (m, 1H, C(4')*H*); 3.86 (dxd, 1H, C(5')*H'*, $^2J_{HH}$=11.55 Hz, $^3J_{HH}$=3.94 Hz); 3.71 (dxd, 1H, C(5')*H''*, $^2J_{HH}$=11.59 Hz, $^3J_{HH}$=2.49 Hz); 0.93-0.81 (3s, 27H, 3x-C-(CH_3)$_3$); 0.11- (-0.04) (m, 18H, 3x-Si-(CH_3)$_2$) ppm.

2′,3′,5′-Tri-*O*-(*tert*-butyldimethylsilyl)-(5-^{13}CH$_3$)-5-methyluridine (**3**)

Compound **2** (7.00 g, 11.9 mmol, 1.00 eq.) was dried in *vacuo* over night before it being dissolved in 70 mL of anhydrous THF and cooled to -80°C. Tetramethylethylenediamine (3.96 mL, 26.2 mmol, 2.20 eq.) was added followed by a stepwise addition of *sec*-butyllithium (42.6 mL, 59.6 mmol, 5.00 eq.). The reaction mixture was stirred at -80°C for 40 minutes under argon atmosphere while ^{13}C-labelled methyl iodide (2.98 mL, 47.7 mmol, 4.00 eq.) was mixed with 25 mL anhydrous THF, which was then added to the reaction mixture in several portions. A white precipitate formed, and the suspension was stirred at -80°C for 30 minutes. The mixture was allowed to warm up to room temperature and was then stirred for another hour until TLC (*n*-Hex/EA: 8/2) confirmed complete conversion of the reaction. The precipitate was filtered, washed with THF and the filtrate evaporated. The residue was dissolved in a little of DCM/MeOH (98/2) and filtered over a pad of silica. After elution of the crude product with the same mixture, all solvents were removed at the rotavapor. The residue was purified by column chromatography (SiO_2, *n*-hex/EA: 95/5-8/2) to obtain pure compound **3** as a yellow solid.

Yield: 5.40 g of a yellow solid (8.97 mmol, 75%);

TLC: (*n*-Hex/EA: 8/2); $R_f = 0.3$;

^{1}H-NMR (400 MHz, DMSO-d_6, 25°C): δ 11.42 (s, 1H, N(3)*H*); 7.42 (m, 1H, C(6)*H*); 5.88 (d, 1H, C(1')*H*, $^3J_{HH}$=7.09 Hz); 4.21 (m, 1H, C(2')*H*); 4.06 (m, 1H, C(3')*H*); 3.93 (m, 1H, C(4')*H*);

3.82 (m, 1H, C(5')*H'*); 3.73 (m, 1H, C(5')*H''*); 1.79 (d, 3H, $^{13}CH_3$, $^1J_{13CH}$=128.65 Hz); 0.93-0.79 (3s, 27H, 3x-C-(CH_3)$_3$); 0.12- (-0.11) (m, 18H, 3x-Si-(CH_3)$_2$) ppm;
^{13}C-NMR (101 MHz, DMSO-d_6, 25°C): δ 12.06 ($^{13}CH_3$) ppm.

(5-$^{13}CH_3$)-5-Methyluridine (**4**)

To a solution of compound **3** (5.40 g, 8.97 mmol, 1.00 eq.) in 60 mL anhydrous THF triethylamino trishydrofluoride (7.46 mL, 44.9 mmol, 5.00 eq.) was added and the yellow solution was stirred over night at room temperature under argon atmosphere. After TLC (DCM/MeOH: 95/5) showed complete conversion, all solvents were evaporated, and the oily residue was dried in *vacuo* for one hour. The crude product was purified twice by column chromatography (SiO_2, DCM/MeOH: 95/5-8/2) to give pure compound **4** as a yellowish oil.
Note: It is necessary to quench the reaction batch by adding quenching buffer prior to column chromatography to avoid a rapid reaction with silica.
Yield: 2.33 g of a yellowish oil (8.97 mmol, 100%);
TLC: (DCM/MeOH: 95/5); R_f = 0.0;
^{1}H-NMR (400 MHz, DMSO-d_6, 25°C): δ 11.27 (br, 1H, N(3)*H*); 7.74 (dxd, 1H, C(6)*H*, $^3J_{13CH}$ =4.31 Hz, $^4J_{HH}$=0.99 Hz); 5.77 (d, 1H, C(1')*H*, $^3J_{HH}$=5.50 Hz); 5.40-4.95 (br, 3H, C(2')O*H*, C(3')O*H*, C(5')O*H*); 4.03 (m, 1H, C(2')*H*); 3.98 (m, 1H, C(3')*H*); 3.82 (m, 1H, C(4')*H*); 3.62 (dxd, 1H, C(5')*H'*, $^2J_{HH}$=12.25 Hz, $^3J_{HH}$=3.43 Hz); 3.54 (dxd, 1H, C(5')*H''*, $^2J_{HH}$=12.25 Hz, $^3J_{HH}$=3.27 Hz); 1.76 (d, 3H, $^{13}CH_3$, $^1J_{13CH}$=128.49 Hz) ppm;
^{13}C-NMR (101 MHz, DMSO-d_6, 25°C): δ 12.20 ($^{13}CH_3$) ppm.

(5-$^{13}CH_3$)-6-D-5-Methyluridine (**5**)

A premixed solution of sodium hydroxide (2.50 g, 14.5 mmol, 2.50 eq.) in 6 mL D_2O was added to a solution of compound **4** (1.50 g, 5.79 mmol, 1.00 eq.) in 20 mL DMSO-d_6. The mixture was stirred for 7 days at 135°C under argon atmosphere until the complete H/D-exchange at position 6 was confirmed by ^{1}H-NMR spectroscopy. All the solvents were evaporated, the residue was suspended in H_2O and neutralized with concentrated HCl. After removing all solvents at the rotavapor, the residue was dissolved in 50 mL of a mixture of DCM/MeOH (8/2) and the solid was filtered off. This procedure was repeated until no product was left in the solid. Again, all solvents were evaporated, the residue was dried in *vacuo* and the crude product was purified by column chromatography (SiO_2, DCM/MeOH: 9/1-1/1) to obtain pure compound **5** as a white foam.
Yield: 1.21 g of a white foam (4.65 mmol, 80%);
TLC: -

^{1}H-NMR (400 MHz, DMSO-d$_6$, 25°C): δ 11.25 (br, 1H, N(3)*H*); 5.77 (d, 1H, C(1')*H*, $^3J_{HH}$=5.59 Hz); 5.51-5.02 (br, 3H, C(2')O*H*, C(3')O*H*, C(5')O*H*); 4.03 (m, 1H, C(2')*H*); 3.97 (m, 1H, C(3')*H*); 3.82 (m, 1H, C(4')*H*); 3.63 (dxd, 1H, C(5')*H'*, $^2J_{HH}$=12.09 Hz, $^3J_{HH}$=3.20Hz); 3.54 (dxd, 1H, C(5')*H''*, $^2J_{HH}$=12.09Hz, $^3J_{HH}$=3.20Hz); 1.76 (d, 3H, ^{13}C*H$_3$*, $^1J_{13CH}$=128.37 Hz) ppm;
^{13}C-NMR (101 MHz, DMSO-d$_6$, 25°C): δ 12.15 (^{13}CH$_3$) ppm.

2′-*O*-(*tert*-Butyldimethylsilyl)-3′,5′-*O*-(di-*tert*-butylsilyl)-(5-^{13}CH$_3$)-6-D-5-methyluridine (**6**)

Compound **5** (1.76 g, 6.76 mmol, 1.00 eq.) was dried under high vacuum at 60 °C overnight before it was suspended in 14 mL absolute DMF and cooled to 0 °C. Di-*tert*-butylsilyl bis(trifluoromethanesulfonate) (2.43 mL, 7.44 mmol, 1.10 eq.) was added dropwise to the suspension and the mixture was stirred at 0 °C under argon atmosphere. After one hour TLC (DCM/MeOH: 92/8) showed a complete conversion and imidazole (2.30 g, 33.8 mmol, 5.00 eq.) was added. After another 5 minutes at 0 °C the solution was allowed to warm up to room temperature and it was stirred for 30 minutes. *Tert.*-butyldimethylsilyl chloride (1.22 g, 8.12 mmol, 1.20 eq.) was added and heated up to 60 °C for 2 hours. After complete conversion of the reaction was confirmed via TLC (*n*-Hex/EA: 6/4) the reaction was quenched with MeOH. The solvent was removed at the rotavapor. The residue was dissolved in ethyl acetate, washed once with saturated sodium bicarbonate solution and trice with saturated sodium chloride solution. The organic layer was dried over sodium sulfate, the filtrate was evaporated to dryness and the crude product was purified by column chromatography (SiO$_2$, *n*-hex/EA: 9/1 - 6/4) to obtain pure compound **6** as a yellowish foam.
Yield: 2.50 g of a yellowish foam (4.86 mmol, 72%);
TLC: (*n*-Hex/EA: 7/3); R_f = 0.5;
^{1}H-NMR (400 MHz, DMSO-d$_6$, 25°C): δ 11.40 (s, 1H, N(3)*H*); 5.66 (d, 1H, C(1')*H*, $^3J_{HH}$=1.08 Hz); 4.38 (m, 1H, C(2')*H*); 4.36 (m, 1H, C(5')*H'*); 4.09 (m, 1H, C(3')*H*); 3.99 (m, 1H, C(5')*H''*); 3.85 (m, 1H, C(4')*H*); 1.79 (d, 3H, ^{13}C*H$_3$*, $^1J_{13CH}$=127.82 Hz); 1.06-0.85 (3s, 27H, 3x-C-(C*H$_3$*)$_3$); 0.12-0.06 (2s, 6H, -Si-(C*H$_3$*)$_2$) ppm;
^{13}C-NMR (101 MHz, DMSO-d$_6$, 25°C): δ 11.93 (^{13}CH$_3$).

2′-*O*-(*tert*-Butyldimethylsilyl)-3′,5′-*O*-(di-*tert*-butylsilyl)-(5-^{13}CH$_3$)-6-D-5-methylcytidine (**7**)

Compound **6** (2.50 g, 4.86 mmol, 1.00 eq.) was dried under high vacuum overnight before it was suspended in 37 mL anhydrous ACN and triethylamine (6.73 mL, 48.6 mmol, 10.0 eq.) and a spatula tip DMAP were added. 2,4,6-Triisopropylbenzenesulfonyl chloride (1.62 g, 5.34 mmol, 1.10 eq.) was added in three portions and the mixture was stirred at room temperature for two hours under argon atmosphere until TLC (*n*-hex/EA: 7/3) confirmed complete conversion. Then 30 mL aqueous ammonia solution (28%) was added, and the solution was

stirred over night at room temperature. After TLC (DCM/MeOH: 9/1) showed full conversion of the intermediate product, all solvents were evaporated, and the residue was dissolved in ethyl acetate and subsequently washed twice with saturated sodium bicarbonate solution. The organic layer was dried over sodium sulfate, the filtrate was evaporated to dryness and the crude product was purified by column chromatography (SiO_2, *n*-Hex/EA: 1/9 - 0/1 → EA/MeOH: 1/0 – 9/1) to obtain pure compound **7** as an ochre foam.

Yield: 1.98 g of an ochre foam (3.85 mmol, 79%);

TLC: (DCM/MeOH: 9/1); $R_f = 0.5$;

^{1}H-NMR (400 MHz, DMSO-d_6, 25°C): δ 7.15 (2s, 2H, -N*H*$_2$); 5.67 (d, 1H, C(1')*H*, $^3J_{HH}$=0.8 Hz); 4.35 (m, 1H, C(5')*H'*); 4.31 (m, 1H, C(2')*H*); 4.08 (m, 1H, C(3')*H*); 3.98 (m, 1H, C(5')*H''*); 3.84 (m, 1H, C(4')*H*); 1.84 (d, 3H, ^{13}C*H*$_3$, $^1J_{13CH}$=128.32 Hz); 1.06-0.85 (3s, 27H, 3x-C-(C*H*$_3$)$_3$); 0.12-0.06 (2s, 6H, -Si-(C*H*$_3$)$_2$) ppm;

^{13}C-NMR (101 MHz, DMSO-d_6, 25°C): δ 12.95 (^{13}CH$_3$) ppm.

N^4-Acetyl-2′-*O*-(*tert*-butyldimethylsilyl)-3′,5′-*O*-(di-*tert*-butylsilyl)-(5-^{13}CH$_3$)-6-D-5-methylcytidine (**8**)

Compound **7** (1.98 g, 3.85 mmol, 1.00 eq.) was dried under high vacuum overnight before it was dissolved in 30 mL anhydrous DMF and a spatula tip DMAP and acetic anhydride (546 µL, 5.78 mmol, 1.50 eq.) were added. The solution was stirred overnight at room temperature under argon atmosphere until TLC (DCM/MeOH: 92/8) showed complete conversion. The reaction was quenched with methanol and all the solvents were evaporated. Afterwards the residue was dissolved in ethyl acetate, washed twice with saturated sodium bicarbonate solution and twice with saturated sodium chloride solution. The organic layer was dried over sodium sulfate, the solvent was removed at the rotation evaporator and the crude product was purified by column chromatography (SiO_2, *n*-hex/EA: 8/2 - 2/8) to gain pure compound **8** as a white foam.

Yield: 1.50 g of a white foam (2.70 mmol, 70%);

TLC: (DCM/MeOH: 92/8); $R_f = 0.7$;

^{1}H-NMR (400 MHz, DMSO-d_6, 25°C): δ 9.79 (s, 1H, N^4*H*); 5.69 (s, 1H, C(1')*H*); 4.45-4.33 (m, 2H, C(5')*H'*, C(2')*H*); 4.16-3.92 (m, 3H, C(5')H'', C(3')*H*, C(4')*H*); 2.27 (s, 3H, -CO-C*H*$_3$);1.98 (d, 3H, ^{13}C*H*$_3$, $^1J_{13CH}$=128.95 Hz); 1.07-0.85 (3s, 27H, 3x-C-(C*H*$_3$)$_3$); 0.17-0.06 (2s, 6H, -Si-(C*H*$_3$)$_2$) ppm;

^{13}C-NMR (101 MHz, DMSO-d_6, 25°C): δ 13.48 (^{13}CH$_3$) ppm.

N[4]-Acetyl-2′-*O*-(*tert*-butyldimethylsilyl)-(5-$^{13}CH_3$)-6-D-5-methylcytidine (**9**)

Compound **8** (1.50 g, 2.70 mmol, 1.00 eq.) was dissolved in 25 mL anhydrous DCM and cooled to 0°C, while a mixture of hydrogen fluoride pyridine (70/30, 270 µL, 10.4 mmol, 3.85 eq.) and anhydrous pyridine (1.74 mL, 21.6 mmol, 8.00 eq.) was prepared and cooled to 0°C. The hydrogen fluoride pyridine mixture was added to the cooled reaction solution and stirred for 3 hours at 0°C under argon atmosphere until TLC-control (DCM/MeOH: 9/1) showed complete conversion. The solution was diluted with DCM, washed once with water, twice with saturated bicarbonate solution and the organic layer was dried over sodium sulfate. After filtration, the solvent evaporated, and the product was co-evaporated with DCM twice. The resulting crude product **9** was used for the next synthesis step without further purification.

Yield: 1.12 g of a white solid (theoretical yield, 2.70 mmol, 100%);

TLC: (DCM/MeOH: 9/1); R_f = 0.5;

^{1}H-NMR (400 MHz, DMSO-d_6, 25°C): δ 9.82 (s, 1H, N^4*H*); 5.69 (d, 1H, C(1')*H*, $^3J_{HH}$=2.22 Hz); 5.25 (t, 1H, C(5')O*H*, $^3J_{HH}$=4.86 Hz); 4.95 (d, 1H, C(3')O*H*, $^3J_{HH}$=5.34 Hz); 4.08 (m, 1H, C(2')*H*); 3.97 (m, 1H, C(3')*H*); 3.92 (m, 1H, C(4')*H*); 3.81 (m, 1H, C(5')*H'*); 3.62 (m, 1H, C(5')*H''*); 2.24 (s, 3H, -CO-CH_3); 1.94 (d, 3H, $^{13}CH_3$, $^1J_{13CH}$=128.67 Hz); 0.87 (s, 9H, -C-(CH_3)$_3$); 0.07 (2s, 6H, -Si-(CH_3)$_2$) ppm;

^{13}C-NMR (101 MHz, DMSO-d_6, 25°C): δ 13.73 ($^{13}CH_3$) ppm.

N[4]-Acetyl-2′-*O*-(*tert*-butyldimethylsilyl)-5′-*O*-[4,4′-(dimethoxytrityl)]-(5-$^{13}CH_3$)-6-D-5-methylcytidine (**10**)

Crude compound **9** (1.12 g, 2.70 mmol, 1.00 eq.) was dissolved in 17 mL anhydrous pyridine and 4,4'-dimethoxytrityl chloride (1.10 g, 3.23 mmol, 1.20 eq.) was added in three portions. The orange solution was stirred over night at room temperature under argon atmosphere until TLC (DCM/MeOH: 95/5) showed complete conversion. After quenching the reaction with MeOH, all solvents were evaporated, and the residue was dissolved in ethyl acetate. Subsequently the solution was washed with aqueous citric acid (5%) twice and saturated sodium bicarbonate solution twice. The organic phase was dried over sodium sulfate, the solvent evaporated, and the crude product purified by column chromatography (SiO_2, *n*-hex/EA: 9/1 - 0/1) to obtain pure compound **10** as a white foam.

Yield: 1.53 g of a white foam (2.13 mmol, 79%);

TLC: (DCM/MeOH: 95/5); R_f = 0.6;

^{1}H-NMR (400 MHz, DMSO-d_6, 25°C): δ 9.80 (s, 1H, N^4*H*); 7.43-7.22 (m, 9H, arom.C*H*); 6.91 (d, 4H, arom.C*H*-C-OCH_3); 5.75 (s, 1H, C(1')*H*); 5.12 (d, 1H, C(3')O*H*, $^3J_{HH}$=5.92 Hz); 4.23-4.06 (m, 3H, C(2')*H*, C(3')*H*, C(4')*H*); 3.74 (s, 6H, 2x-OCH_3); 3.32 (m, 2H, C(5')H_2); 2.25 (s,

3H, -CO-CH_3);1.49 (d, 3H, ^{13}CH_3, $^1J_{13CH}$=128.27 Hz); 0.87 (s, 9H, -C-(CH_3)$_3$); 0.09 (2s, 6H, -Si-(CH_3)$_2$) ppm;
^{13}C-NMR (101 MHz, DMSO-d_6, 25°C): δ 13.35 (^{13}CH$_3$) ppm.

N^4-Acetyl-2′-*O*-(*tert*-butyldimethylsilyl)-5′-*O*-[4,4′-(dimethoxytrityl)]-(5-^{13}CH$_3$)-6-D-5-methylcytidine-3'-*O*-(2-cyanoethyl-*N,N*-diisopropylphosphoramidite) (**11**)

Compound **10** (1.53 g, 2.13 mmol, 1.00 eq.) was dried overnight in *vacuo* and then dissolved in 15 mL anhydrous THF. *N,N*-Diisopropylethylamine (1.86 mL, 10.6 mmol, 5.00 eq.) and 2-cyanoethyl-*N,N*-diisopropylchlorophosphoramidite (714 µL, 3.20 mmol, 1.50 eq.) were added simultaneously and the solution was stirred over night at room temperature under argon atmosphere. After TLC (DCM/MeOH: 95/5 + 1% NEt$_3$) confirmed complete conversion, the reaction was quenched with MeOH, diluted with DCM and washed with half-saturated sodium bicarbonate solution twice. The organic layer was dried over sodium sulfate, the solvent evaporated, and the residue purified by column chromatography twice (SiO$_2$, *n*-hex/EA: 7/3 - 2/8+1% triethylamine; SiO$_2$, EA +1% triethylamine) to obtain pure compound **11** as a white foam consisting of two diastereomers.
Yield: 1.51 g of a white foam (1.64 mmol, 77%);
TLC: (DCM/MeOH + 1% NEt$_3$: 95/5); R_f = 0.6;
^{1}H-NMR (400 MHz, DMSO-d_6, 25°C): δ 9.81 (s, 1H, N^4*H*); 7.43-7.22 (m, 9H, arom.C*H*); 6.93-6.87 (m, 4H, arom.C*H*-C-OCH$_3$); 5.88 (d, 1H, C(1')*H*, $^3J_{HH}$=4.24 Hz); 4.49-4.39 (m, 1H, C(2')*H*); 4.29-4.14 (m, 2H, C(3')*H*, C(4')*H*); 3.74 (s, 6H, 2x-OCH_3); 3.81-3.58 (m, 1H, -P-O-CH$_2$-C*H*'$_2$-); 3.65-3.43 (m, 3H, -P-O-CH$_2$-C*H*''$_2$-, 2x-N-C*H*-(CH$_3$)$_2$); 3.41-3.24 (m, 2H, C(5')H_2); 2.76 (m, 1H, -P-O-C*H*'$_2$-CH$_2$-); 2.54 (m, 1H, -P-O-C*H*''$_2$-CH$_2$-); 2.24 (s, 3H, -CO-CH_3);1.51 (d, 3H, ^{13}CH_3, $^1J_{13CH}$=128.79 Hz); 1.12-0.90 (m, 12H, 2x-N-CH-(CH_3)$_2$); 0.85 (s, 9H, -C-(CH_3)$_3$); 0.07 (2s, 6H, -Si-(CH_3)$_2$) ppm;
^{13}C-NMR (101 MHz, DMSO-d_6, 25°C): δ 13.25, 13.09 (^{13}CH$_3$) ppm;
^{31}P-NMR (162 MHz, DMSO-d_6, 25°C): 148.83 (s); 148.51 (s) ppm;
ESI-MS: calc.: 917.4464, found: 918.4513 [M+H]$^+$, 940.4329 [M+Na]$^+$, 956.4067 [M+K]$^+$.

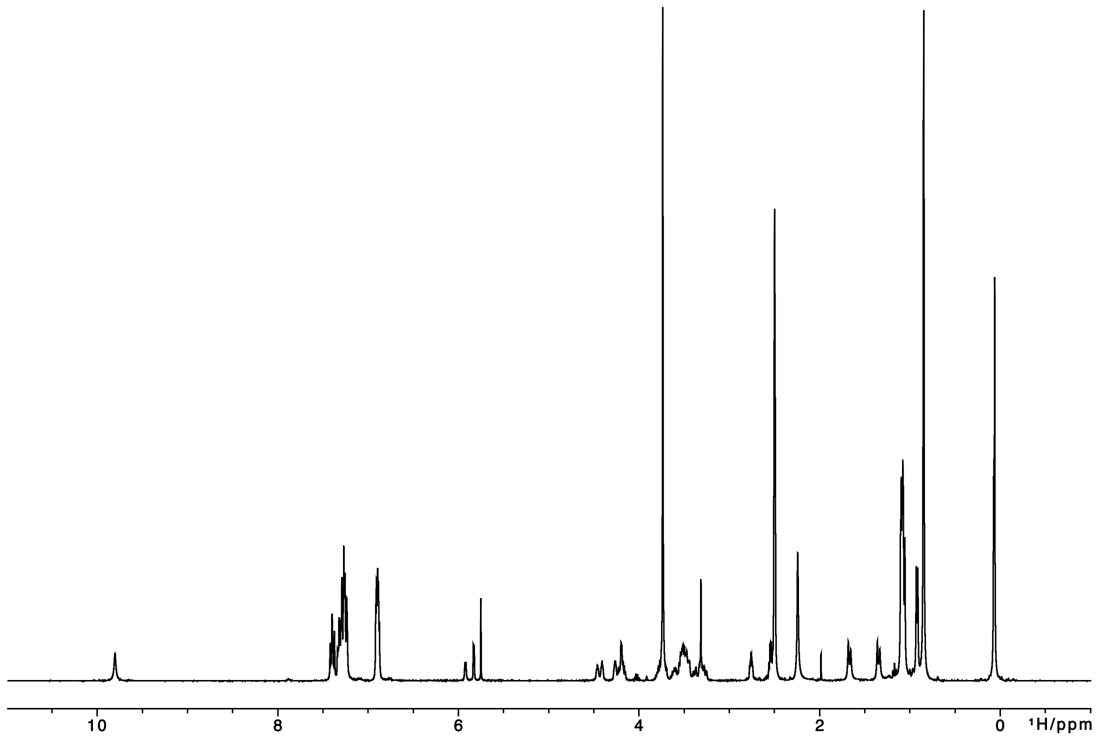

Supporting Figure 1. ^{1}H-NMR of (5-$^{13}CH_3$)-6-D-5-methylcytidine phosphoramidite **11** (400 MHz, DMSO-d_6, 25°C).

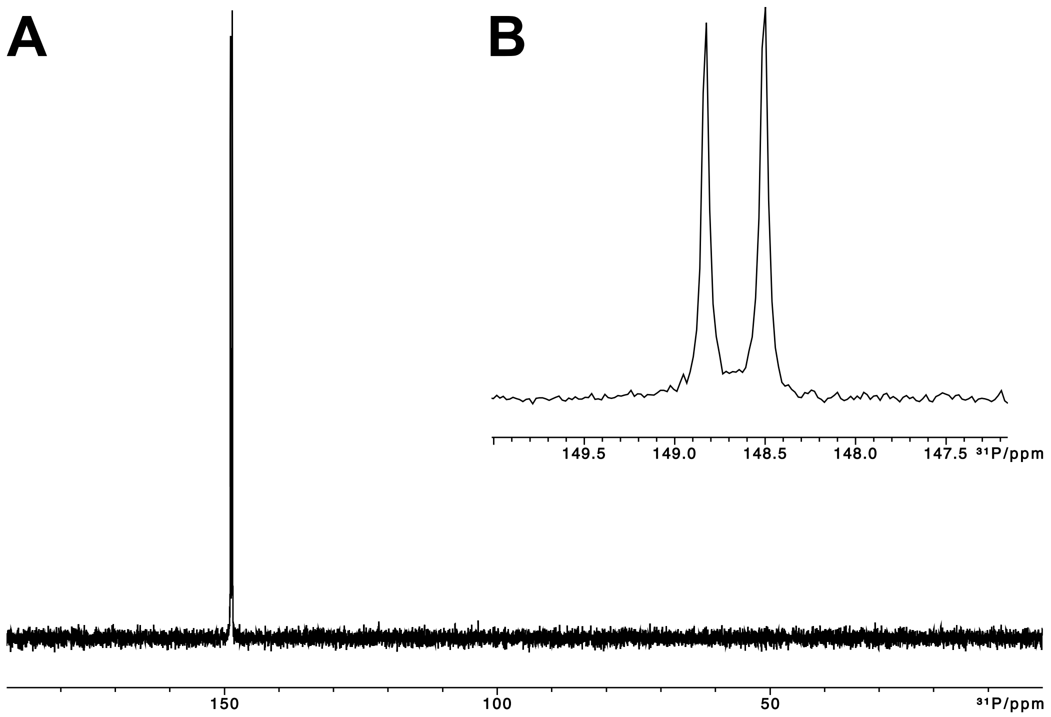

Supporting Figure 2. ^{31}P-NMR of (5-$^{13}CH_3$)-6-D-5-methylcytidine phosphoramidite **11** (162 MHz, DMSO-d_6, 25°C). **A)** Full spectral width and **B)** zoom on the ^{31}P resonances of **11**.

RNA synthesis

Standard 2´-*O*-TBDMS RNA phosphoramidites (rA^{Ac}, rC^{Ac}, rG^{Ac} and rU, *Chemgenes*, USA) were used in combination with in-house synthesized labeled (5-$^{13}CH_3$)-6-D-5-methylcytidine, (3-$^{13}CH_3$)-5-D-6-^{13}C-3-methyluridine, 8-^{13}C-adenosine, 8-^{13}C-guanosine, 5-D-6-^{13}C-uridine, 5-D-6-^{13}C-cytidine, 1-^{15}N-guanosine, 1,3-$^{15}N_2$-uridine, and unlabeled N^4-methyl-2'-*O*-methyl-cytidine RNA phosphoramidites. A controlled pore glass (CPG) RNA solid support (1000 Å pore size, *Chemgenes*, USA) with an average loading of 40 µmol g^{-1} was used to synthesize the RNAs on an ABI 391 PCR Mate or a *K&A Laborgeräte* H6 using a self-written synthesis cycle. Amidite (0.1M) and activator (5-benzylthio-1*H*-tetrazole, 0.25 M) solutions were dried over freshly activated molecular sieves (3Å) for at least 48 hours before starting the solid phase synthesis, for which following reagent mixtures were used: *Cap A*: acetic anhydride/lutidine/tetrahydrofuran 1/1/8, v/v/v. *Cap B*: tetrahydrofuran/N-methylimidazole 86/16 v/v. *Oxidation solution*: 500 mg iodine dissolved in a mixture of 70 mL THF, 20 mL pyridine and 10 mL water. *Detritylation solution*: 4% dichloroacetic acid in anhydrous toluene or in dichloroethane.

Following the completion of the RNA synthesis, the solid support was dried in *vacuo* for 30 minutes. Then *standard alkaline deprotection* was performed: 1 mL aqueous methylamine solution (40%) and 1 mL aqueous ammonia solution (28-30%) were added to the solid support and the tube was shaken vigorously and incubated at 37°C for 5 hours. The solid support was filtered, then washed three times with a mixture of THF/water (1/1) and the liquid phase evaporated to dryness. The residue was dried in *vacuo* for at least 1 hour before the *2´-O-TBDMS deprotection* was carried out: The residue from the last step was dissolved in 300 µL anhydrous DMSO and 375 µL triethylamine trihydrofluoride was added. Then, the protection mixture was incubated at 37°C for at least 16 hours. 3 mL Quenching buffer (*ChemGenes*, USA) were added and the solution directly applied to a HiPrep 26/10 desalting column (*GE Healthcare*, Austria) using a ÄKTA Start system (*GE Healthcare*, Austria). The crude RNA was eluted using HPLC grade water and the RNA containing fractions (UV detection at 254 nm) were collected in a 50 mL round bottom flask. After the solvent was evaporated, the crude RNA again dissolved in 1 mL HPLC grade water and transferred in a 1.5 mL *Eppendorf* tube. The quality of the crude RNAs was checked via anion exchange chromatography on an analytical Dionex DNAPac PA-200 column (4x250 mm; *Eluent A*: 25 mM Tris.HCl, 10 mM sodium perchlorate, 20% acetonitrile, pH 8.0; *Eluent B*: 25 mM Tris.HCl, 600 mM sodium perchlorate, 20% acetonitrile, pH 8.0) and at elevated temperature (80°C). The RNA sequences were purified in a single run by applying the crude RNA on a preparative Dionex DNAPac PA-200 column (22x250 mm, eluents as before). The fractions with the desired RNA were combined and ACN was evaporated before it was loaded on a C18 SepPak cartridge (*Waters*, Austria) to remove HPLC buffer salts. The RNA sodium salt form was then eluted

from the C18 column with water/acetonitrile (1/1, v/v), evaporated to dryness, dissolved again in 1 mL HPLC grade water and transferred in a 1.5 mL *Eppendorf* tube for quality (analytical HPLC as before) resp. concentration determination and mass spectrometric analysis (LC-ESI. Mass spectrometry).

Sample concentrations were determined via UV absorption at 260 nm on a NanoPhotometer (*Implen*) or in a UV cuvette (1 cm light-path) via a *Varian* Cary 100 Scan UV/Vis spectrometer.

RNA LC-ESI mass spectrometry

The synthesized RNAs were analyzed on Finnigan LCQ Advantage MAX ion trap instrumentation connected to a Thermo Scientific UHPLC (components: Ultimate 3000 RS Pump, Ultimate 3000 RS Autosampler, Ultimate 3000 RS Column Compartment, Ultimate 3000 Diode Array Detector). RNA mass spectra were acquired in the negative-ion mode with a potential of -4 kV applied to the spray needle (capillary voltage: -23V, capillary temperature: 270°C). LC: 1 µL of the dissolved RNA in 29 µL of 20 mM ethylenediaminetetraacetic acid (EDTA) solution; average injection volume: 30 µL; column: Waters xBridge C18 2.5 µm column (1.0 × 50 mm) at 30°C; flow rate: 100 µL/min; *Eluent A*: 8.6 mM triethylamine (TEA), 100 mM 1,1,1,3,3,3-hexafluoroisopropanol in H_2O (pH 8.0); *Eluent B*: methanol; gradient: 0– 100% B in A within 30 min; UV detection was carried out at 260/280 nm. The correct assembly of the synthesized RNAs was confirmed by the mass data and the RNA samples were subsequently lyophilized.

Supporting Table 1. Overview of the synthesized RNAs with the found and calculated mass. $^{13}C/^{2}H$ labeled nucleotides are highlighted in red.

No.	nts	sequence	mass found / AMU	mass calc. / AMU
**1a**, C1407	44	5´- GGA CCG m^{4}CmCC GUC ACA CCG CUU CGG CGG UGA AGU CGm^{3}U AAC GGU CC -3´	14170.79	14171.43
**1b**, m^{5}C1407	44	5´- GGA CCG m^{4}CmCC GUm^{5}C ACA CCG CUU CGG CGG UGA AGU CGm^{3}U AAC GGU CC -3´	14185.09	14185.46
**2a**, C1407	27	5´- GGC GUC ACA CCU UCG GGU GAA GUC GCC -3´	8658.88	8659.13
**2b**, m^{5}C1407	27	5´- GGC GUm^{5}C ACA CCU UCG GGU GAA GUC GCC -3´	8672.91	8673.16

NMR spectroscopy

RNA samples were lyophilized as sodium salts and were either dissolved in 280 µL NMR buffer (15 mM sodium phosphate, 25 mM NaCl, 0.1% NaN_3, pH 6.5, 10 % or 100% D_2O) for Shigemi tubes or 440 µL NMR buffer for standard 5 mm *Norell* borosilicate NMR tubes giving 0.5 to 1.2 mM sample concentrations. All NMR experiments were conducted on a Bruker 700 MHz Avance 4 Neo spectrometer or a Bruker 600 MHz Avance 4 Neo spectrometer, both equipped with a Prodigy TCI probe. Experiments were run at the temperatures indicated (10°C and 25°C).

For the imino proton spectra the IBS imino pulse program (part of the NMRlib 2.0 package, available from IBS Grenoble) was used with selective excitation of imino protons. The 1D spectrum was acquired with 512 scans and an interscan delay of 300 ms giving an experiment time of ca. 4 min. Signal assignments are based on ^{1}H-^{1}H-NOESY, ^{1}H-^{13}C-HSQC, and ^{1}H-^{15}N-HSQC experiments.

Acquisition and analysis of relaxation dispersion experiments

^{1}H CPMG relaxation dispersion NMR

For the proton CPMG relaxation dispersion experiments the following ^{1}H CPMG frequencies (ν_{CPMG}) were used: 100, 100, 200, 400, 600, 800, 1200, 1600, 2000, 2200, 2200, 2400, 2600, 2800, 3000, 3200, 3400, 3600, 3600, 3800 and 4000 Hz. The constant time relaxation delay T_{relax} was 20 ms for all experiments. 1024*64 complex data points were recorded at 600 MHz and 700 MHz proton resonance frequency with a spectral width of 10 (10) ppm in the proton (carbon) dimension centered at 4.7 (139) ppm. The number of scans was 128, and the interscan delay was set to 1.0 s, yielding a total experimental time of 34 hrs.

^{13}C CPMG relaxation dispersion NMR

For the carbon CPMG relaxation dispersion experiments the following ^{13}C CPMG frequencies (ν_{CPMG}) were used: 100, 200, 200, 300, 400, 500, 500, 600, 700, 800, 900, 900, 1000 Hz. The constant time relaxation delay T_{relax} was 20 ms for all experiments. 1024*64 complex data points were recorded at 150 MHz and 176 MHz carbon resonance frequency with a spectral width of 10 (10) ppm in the proton (carbon) dimension centered at 4.7 (139) ppm. The number of scans was 160, and the interscan delay was set to 1.0 s yielding a total experimental time of 45 hrs.

Spectral processing was performed using the *nmrPipe* and *nmrDraw* software package.[1] Peak integration was performed using *nmrDraw*, and effective relaxation rates were determined as:

$$R_{2,\mathrm{eff}} = -\frac{1}{T_{\mathrm{relax}}} ln\left(\frac{I}{I_0}\right)$$

where I is the intensity at a given ν_{CPMG}, and I_0 is the reference intensity with the T_{relax} set to 0. Uncertainties in relaxation rates (σ_{R2}) were calculated from the repeat experiments at ν_{CPMG}

values of 100, 2200 and 3600 Hz for ^{1}H dispersions and 200, 500 and 900 Hz for ^{13}C dispersions as outlined below.

Analysis of relaxation dispersion experiments

The relaxation dispersion data were analyzed by fitting the Carver-Richards equation for a two-state exchange process to the experimental data to extract exchange-rate constants (k_{ex}) and populations (p_{GS} and p_{ES} , with $p_{\mathrm{GS}} = 1 - p_{\mathrm{ES}}$) pertaining to the exchange process, as well as chemical-shift differences ($\Delta\omega$) between the two states.[2] This was done either *i)* in a per-residue manner, fitting the the ^{1}H and ^{13}C relaxation dispersion data (*i.e.* four dispersion profiles for each residue) assuming a single, residue-specific value for k_{ex} and p_{ES} or *ii)* in a global manner, fitting the the ^{1}H and ^{13}C relaxation dispersion data for a group of residues, assuming a common value for k_{ex} and p_{ES}, but allowing for residue-specific values of the chemical-shift differences between the two states.

The fitting procedure was performed by minimizing the chi-squared target function

$$\chi^2 = \sum_n \frac{\left(R_{2,\mathrm{eff}}^{exp}(n) - R_{2,\mathrm{eff}}^{calc}(n)\right)^2}{(\sigma_{R2}\ (n))^2}$$

where $R_{2,\mathrm{eff}}^{\mathrm{exp}}(n)$ and $R_{2,\mathrm{eff}}^{\mathrm{calc}}(n)$ are the experimental and calculated effective rate constants at data points n (*i.e.,* at all ν_{CPMG} and residue(s) included in the fit). Values of σ_{R2}, assumed to be constant within each dispersion profile, were calculated from the repeat experiments as

$$\sigma_{R2}\ (n) = \sqrt{\frac{\sum_{j=1}^{N} \sigma_j^2\ (n_{\mathrm{j}} - 1)}{\sum_{i=1}^{N} (n_{\mathrm{j}} - 1)}}$$

where N is the number of ν_{CPMG} values at which repeat experiments were performed (N = 3 in our case) in n_{j} replicates (n_{j} = 2 in our case), and σ_j is the standard deviation in these replicates.[3]

Uncertainties in the exchange parameters derived from global fits were estimated *via* a Monte Carlo (MC) approach, in which 100 synthetic datasets were generated using the exchange parameters obtained by the global fits described above, along with the experimental error in the relaxation rates (σ_{R2}). Data fitting was repeated for all datasets; errors quoted in the paper and in the SI are standard deviations in fitted exchange parameters that were obtained in this procedure.

In the first step, the experimental data of all residues (including the A-site and the m^{3}U bulge) but excluding the negative controls for the base paired residues A3, U42 and the UUCG tetraloop (U20, U21) in RNAs **1a** and **1b** were included in a global fitting procedure to a common exchange process. To identify residues for which the experimental data were not

adequately represented by this process, Akaike's Information Criterion corrected for small sample sizes (AICc) was calculated as

$$\mathrm{AICc} = \chi^2 + 2k + (2k(k+1))/(n-k-1)$$

where k is the overall number of parameters that are adjusted in the fitting process and n is the number of data points, as defined above.[4] **Supporting Tables 2 and 3** summarize the selection procedure for RNAs **1a** and **1b**. For both RNAs, AICc values improved (decreased) when both residues in the m^3U bulge (m^3U1498 and A1499) were excluded from the global fit, suggesting unrelated conformational exchange processes for the m^5C and m^3U bulges.

Supporting Table 2. Model selection for 44 nt RNAs **1a** and **1b**.

	RNA 1a			RNA 1b		
	**AICc**	**k_{ex} / s^{-1}**	**p_{ES} / %**	**AICc**	**k_{ex} / s^{-1}**	**p_{ES} / %**
all residues [a)]	2334.4	6206	3.6	2338.8	4598	6.1
excl. m^3U1498 [b)]	2370.5	6167	5.5	2373.4	4744	6.3
excl. A1499 [c)]	2342.2	5749	4.6	2280.3	4666	7.5
excl. m^3U1498+A1499 [d)]	2157.5	5711	4.1	2265.6	4080	4.3

AICc values and fitting results (k_{ex} and p_{ES}) obtained in global fits of [a)] RNA **1a**: U1406, C1407, C1409, U1490, G1491, A1492, A1493, G1494, U1495, m^3U1498, A1499; RNA **1b**: U1406, C1409, U1490, G1491, A1492, A1493, G1494, U1495, m^3U1498, A1499. [b)] residues [a)] but excluding m^3U1498; [c)] residues [a)] but excluding A1499; [d)] residues [a)] but excluding m^3U1498 and A1499.

Fitting distinct exchange processes to the m^3U bulge (m^3U1498 and A1499) and the A-site (all other residues), each characterized by its own k_{ex} and p_{ES} values, and estimating the uncertainties obtained by the Monte Carlo approach described above, revealed different exchange rates for the two segments, with k_{ex} in the A-site < k_{ex} in the m^3U bulge in both RNAs **1a** and RNA **1b** (**Supporting Table 3**). In addition, m^5C1407 methylation reduced k_{ex} in the A-site, from 5706 ± 458 s^{-1} to 4023 ± 339 s^{-1} (RNA **1a** *vs.* RNA **1b**). Regarding populations, differences or trends could not be reliably evaluated due to large uncertainties in the extracted p_{ES} values.

Supporting Table 3. Fitting results and reduced chi-squared values (χ^2_{red}) obtained from global fits of the ^1H and ^{13}C relaxation dispersion data for 44 nt RNAs **1a** and **1b**, assuming distinct exchange processes (k_{ex} and p_{ES} values) for the A-site and the m^3U bulge (uncertainties given in brackets).

	RNA 1a			RNA 1b		
	**k_{ex} / s^{-1}**	**p_{ES} / %**	χ^2_{red}	**k_{ex} / s^{-1}**	**p_{ES} / %**	χ^2_{red}
A-site [a)]	5706 (458)	4.1 (1.4)	3.39	4023 (339)	4.4 (1.9)	4.10
m^3U bulge [b)]	8932 (875)	6.7 (4.1)	2.02	8460 (1255)	8.5 (4.3)	2.48

Residues included [a)] RNA **1a**: U1406, C1407, C1409, U1490, G1491, A1492, A1493, G1494, U1495; RNA **1b**: U1406, C1409, U1490, G1491, A1492, A1493, G1494, U1495; [b)] m^3U1498, A1499.

While the ^{1}H relaxation dispersion profiles for RNAs **1a** and **1b** displayed considerable scattering, in particular for residues with $R_{2,\mathrm{eff}}$ values exceeding 100 s^{-1}, such as G1491 or G1494 (**Supporting Figures 3 and 4**), for which signals of low intensity ratios (I/I_0 < 0.14) were observed at the lowest CPMG frequencies even with T_{relax} set to 20 ms, the dispersion profiles of the smaller 27 nt RNA constructs **2a** and **2b** showed less scattering, along with lower transverse relaxation rates (**Supporting Figures 5 and 6**). Global fits of the dispersion profiles of all residues in **2a** (U1406, C1407, C1409, U1490, G1491, A1492, A1493, G1494, U1495) and in **2b** U1406, A1408, C1409, A1410, U1490, G1491, A1492, A1493, G1494, U1495) yielded the values for the exchange parameters k_{ex} and p_{ES} shown in **Supporting Table 4**. Compared to RNAs **1a** and **1b**, Monte Carlo-derived uncertainties in k_{ex} and p_{ES} were smaller and χ^2_{red} values improved. The extracted ^{1}H and ^{13}C chemical shift differences ($\Delta\omega$) between states are shown in **Supporting Table 5**.

Supporting Table 4. Fitting results (k_{ex} and p_{ES}) and χ^2_{red} values for the A-site in 27 nt RNAs **2a** and **2b**, obtained from global fits of the ^{1}H and ^{13}C relaxation dispersion data (MC-derived uncertainties in brackets).

	RNA 2a			RNA 2b		
	k_{ex} / s^{-1}	p_{ES} / %	χ^2_{red}	k_{ex} / s^{-1}	p_{ES} / %	χ^2_{red}
A-site [a)]	5492 (241)	3.2 (0.7)	2.0	3480 (139)	3.1 (0.2)	1.4

Residues included [a)] RNA **2a**: U1406, C1407, C1409, U1490, G1491, A1492, A1493, G1494, U1495; RNA **2b**: U1406, A1408, C1409, A1410, U1490, G1491, A1492, A1493, G1494, U1495.

Supporting Table 5. Chemical shift differences ($\Delta\omega$) extracted from global fits for 27nt RNAs **2a** and **2b** (MC-derived uncertainties in brackets).

	RNA 2a	$\Delta\omega$ (^{13}C) / ppm		RNA 2b	$\Delta\omega$ (^{13}C) / ppm
residue	$\Delta\omega$ (^{1}H) / ppm		residue	$\Delta\omega$ (^{1}H) / ppm	
U1406	0.21 (0.05)	0.83 (0.17)	U1406	0.17 (0.04)	1.27 (0.17)
C1407	0.36 (0.10)	0.51 (0.28) [a)]	A1408	0.19 (0.03)	0.03 (0.22) [a)]
C1409	0.22 (0.05)	0.48 (0.21) [a)]	C1409	0.23 (0.03)	0.60 (0.16)
U1490	0.20 (0.05)	0.27 (0.18) [a)]	A1410	0.33 (0.04)	0.44 (0.21) [a)]
G1491	0.21 (0.06)	1.24 (0.20)	U1490	0.18 (0.05)	0.28 (0.18) [a)]
A1492	0.24 (0.05)	1.32 (0.19)	G1491	0.24 (0.03)	1.51 (0.17)
A1493	0.32 (0.08)	0.50 (0.21) [a)]	A1492	0.26 (0.03)	1.67 (0.21)
G1494	0.41 (0.09)	0.79 (0.17)	A1493	0.40 (0.05)	0.62 (0.12)
U1495	0.37 (0.07)	1.82 (0.33)	G1494	0.63 (0.10)	1.13 (0.16)
			U1495	0.50 (0.07)	2.44 (0.38)

[a)] Relaxation dispersion profiles with $\Delta R_{2,\mathrm{eff}}$ < 2.0 s^{-1}.

For all residues in the A-site, the validity of assuming a common exchange process was evaluated by systematically excluding each residue from the global fit, one at a time, and assessing *i)* the AICc criterion and *ii)* the effect on the extracted exchange parameters k_{ex} and p_{ES} and (**Supporting Table 6**). For both RNAs **2a** and **2b**, omitting any single residue from the global fit (and fitting it per-residue) did not alter the exchange parameters k_{ex} and p_{ES} within uncertainties, nor did AICc values improve (compare RNAs **1a** and **1b**, **Supporting Table 2**). This indicated that for the A-site in RNAs **2a** and **2b** *i)* the experimental relaxation dispersion data were adequately represented by a common exchange process and *ii)* including dispersion profiles in the global fitting procedure with exchange contributions ($\Delta R_{2,\mathrm{eff}}$ = max($R_{2,\mathrm{eff}}$) − min($R_{2,\mathrm{eff}}$)) < 2.0 s^{-1} (i.e., the ^{13}C dispersion profiles of C1407, A1408, C1409, A1410, U1490, A1493) did not affect the extracted exchange parameters k_{ex} and p_{ES}; for these nuclei the ^{13}C chemical shift differences were small, $\Delta\omega \lesssim 0.5$ ppm (see **Supporting Table 5**), in accordance with the observed small $\Delta R_{2,\mathrm{eff}}$ values.

Supporting Table 6. Evaluation of the global fitting procedure for 27 nt RNAs **2a** and **2b**.

RNA 2a	AICc	k_{ex} / s^{-1}	p_{ES} / %	RNA 2b	AICc	k_{ex} / s^{-1}	p_{ES} / %
all residues [a)]	1240.7	5492 (241)	3.2 (0.7)	all residues [a)]	1030.5	3480 (139)	3.1 (0.2)
excl. U1406 [b)]	1323.4	5779 (229)	3.4 (0.6)	excl. U1406 [b)]	1028.3	3563 (165)	3.3 (0.4)
excl. C1407 [b)]	1255.8	5260 (200)	3.2 (0.5)	excl. A1408 [b)]	1006.1	3605 (164)	3.5 (0.5)
excl. C1409 [b)]	1252.8	5251 (187)	3.2 (0.4)	excl. C1409 [b)]	993.0	3601 (132)	3.2 (0.4)
excl. U1490 [b)]	1231.6	5375 (218)	3.5 (0.5)	excl. A1410 [b)]	1009.5	3481 (218)	3.4 (0.4)
excl. G1491 [b)]	1302.3	5370 (251)	3.1 (0.4)	excl. U1490 [b)]	1036.9	3622 (100)	3.4 (0.5)
excl. A1492 [b)]	1248.7	5336 (250)	3.1 (0.5)	excl. G1491 [b)]	1001.4	3569 (124)	3.3 (0.3)
excl. A1493 [b)]	1226.4	5222 (178)	3.1 (0.4)	excl. A1492 [b)]	1019.3	3489 (140)	3.2 (0.2)
excl. G1494 [b)]	1235.2	5666 (231)	3.1 (0.5)	excl. A1493 [b)]	1018.6	3499 (162)	3.3 (0.3)
excl. U1495 [b)]	1250.2	5363 (343)	2.8 (0.6)	excl. G1494 [b)]	992.9	3389 (137)	2.9 (0.3)
				excl. U1495 [b)]	1030.3	3298 (110)	2.9 (0.3)

AICc values and fitting results (k_{ex} and p_{ES}) obtained from global fits of [a)] RNA **2a**: U1406, C1407, C1409, U1490, G1491, A1492, A1493, G1494, U1495; RNA **2b**: U1406, A1408, C1409, A1410, U1490, G1491, A1492, A1493, G1494, U1495; [b)] residue excluded from global fit and fitted on per-residue basis.

Melting curve studies and determination of thermodynamic parameters

The UV absorbance measurements were conducted using a *Varian* Cary 100 UV-visible spectrometer, which is equipped with a multiple cell holder and a *Peltier* temperature control device. Data points were recorded at two distinct wavelengths (250 nm and 260 nm) as a function of temperature, with the blank value detected at 320 nm. The data points were collected in 0.5°C increments during three sets of heating and cooling cycles to verify the essential reversibility for a monomolecular melting transition, with a heating and cooling rate of 0.7°C/min (20°C → 95°C → 10°C → 95°C → 10°C → 95°C).

Three amounts of the nucleic acid stock solution were lyophilized, yielding a final concentration of ca. 2 µM, 5 µM and 8 µM, respectively. This was achieved by dissolving the lyophilized oligonucleotide in 800 µL of the corresponding melting curve buffer (10 mM Na_2HPO_4, 150 mM NaCl, pH=7.0). The solution was subsequently mixed and transferred into a 1 cm quartz cuvette. Following the degassing of the sample by sonication to remove any dissolved air, the solutions were overlaid with dimethyl polysiloxane to prevent variation of the concentration by solvent evaporation. T_m values were determined by calculating the first derivative, usually from data of the second cooling ramp.

Thermodynamic data were calculated using the melting curves of the RNAs with a concentration of ca. 5 µM according to Breslauer and co-workers.[5] Briefly, the association grade α was calculated by aligning two tangents, one for the lower part and one for the upper part of the hyperchromicity (**Supporting Figure 8**). Using the linear regression equation, α can be obtained with the following formula:

$$\alpha = \frac{\mathrm{up} - \mathrm{hyp}}{\mathrm{up} - \mathrm{low}} \tag{1}$$

After calculating α, the resulting graph (α *vs.* T, **Supporting Figure 8**) is fitted with the following formula to obtain values for m_1 and m_2:

$$\alpha = \frac{1}{1 + e^{\frac{m_1 - (T \cdot m_2)}{1.9858 \cdot T}}} \tag{2}$$

The thermodynamic parameters are calculated with the fitted values of m_1 and m_2:

$$\Delta H^0 = \frac{m_1}{1000};\ \Delta S^0 = m_2;\ \Delta G^0 = \Delta H^0 - T \cdot \Delta S^0 \tag{3}$$

Error limits for ΔG^0, ΔH^0 and ΔS^0 are determined as the standard deviation of three independent measurements. All calculations and illustrations of the UV melting experiments were performed in OriginPro 2023.

Supporting Table 7. Melting points and thermodynamic parameters from UV melting curve analyses.

sequence name	T_m / °C	error	ΔG / kcal mol^{-1}	error	ΔH/ kcal mol^{-1}	error	ΔS / cal K^{-1} mol^{-1}	error
ES mimic C1407	87.57	0.10	-21.7	0.5	-127	3	-352	7.4
ES mimic m^5C1407	89.33	0.10	-24.7	0.6	-141	3	-390	9.4
A-site m^5C1407	74.96	0.10	-11.1	0.1	-79	1	-227	2.0
A-site C1407	74.43	0.10	-10.6	0.1	-76	1	-218	1.9

Supporting Figure 3. ^{1}H and ^{13}C CPMG relaxation dispersion of RNA **1a**. **A)** ^{1}H relaxation dispersion profiles. **B)** ^{13}C relaxation dispersion profiles. **C)** Secondary structure and stable isotope labeling pattern of RNA **1a**. Red data points 600 (150) MHz ^{1}H (^{13}C) frequency, blue data points 700 (176) MHz ^{1}H (^{13}C) frequency. Per-residue fits (A3, U20, U21, U42, negative controls) or global fits (for the A-site and the m^{3}U bulge, see **Supporting Table 3** for details) are shown as a full-drawn line in the respective color.

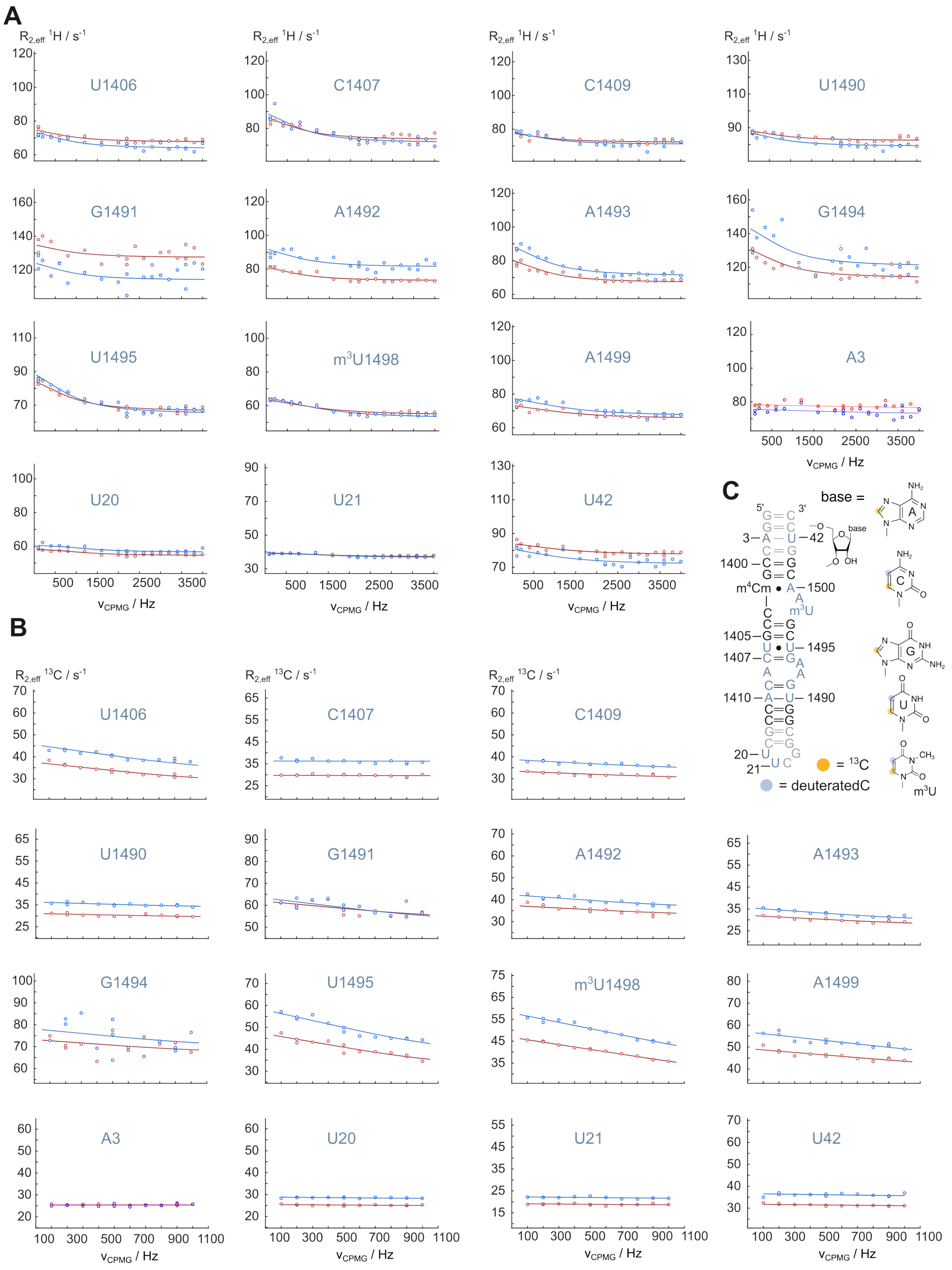

Supporting Figure 4. ^1H and ^{13}C CPMG relaxation dispersion of RNA **1b**. **A)** ^1H relaxation dispersion profiles. **B)** ^{13}C relaxation dispersion profiles. **C)** Secondary structure and stable isotope labeling pattern of RNA **1b**. Red data points 600 (150) MHz ^1H (^{13}C) frequency, blue data points 700 (176) MHz ^1H (^{13}C) frequency. Per-residue fits (A3, U20, U21, U42, negative controls) or global fits (for the A-site and the m^{3}U bulge, see **Supporting Table 3** for details) are shown as a full-drawn line in the respective color.

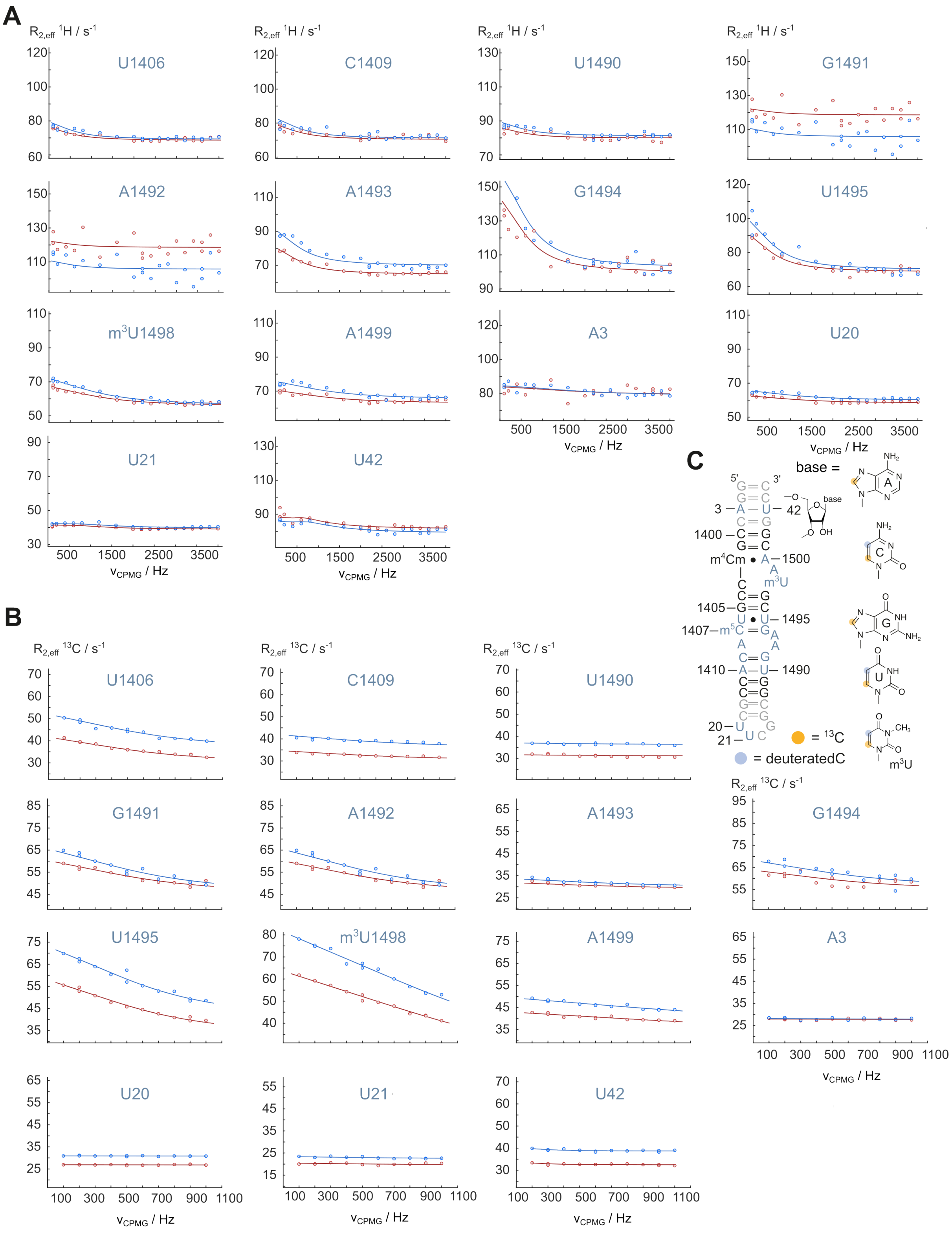

Supporting Figure 5. ^{1}H and ^{13}C CPMG relaxation dispersion of RNA **2a**. **A)** ^{1}H relaxation dispersion profiles. **B)** ^{13}C relaxation dispersion profiles. **C)** Secondary structure and stable isotope labeling pattern of RNA **2a**. Red data points 600 (150) MHz ^{1}H (^{13}C) frequency, blue data points 700 (176) MHz ^{1}H (^{13}C) frequency. Global fits are shown as a full-drawn line in the respective color.

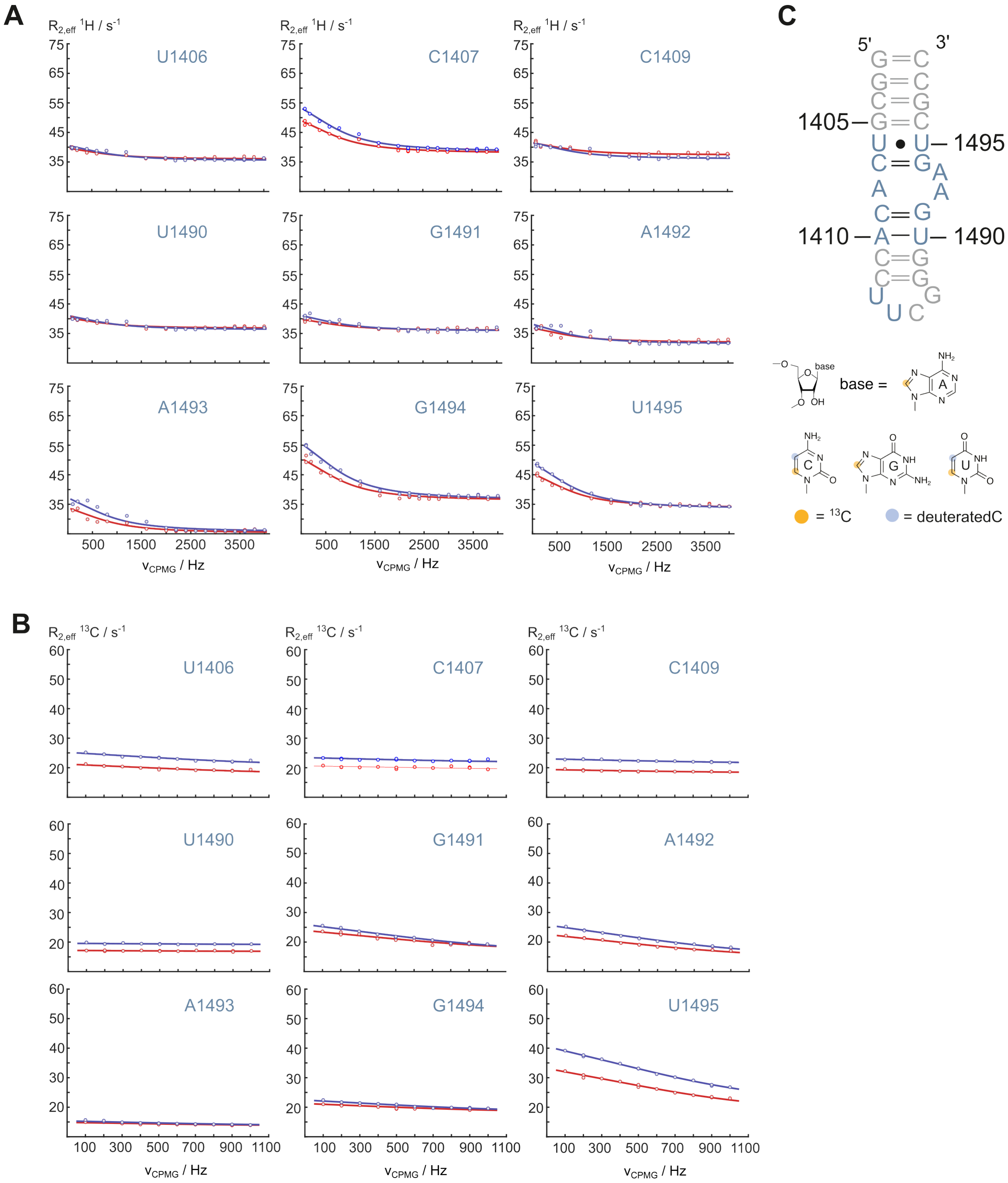

Supporting Figure 6. ^{1}H and ^{13}C CPMG relaxation dispersion of RNA **2b**. **A)** ^{1}H relaxation dispersion profiles. **B)** ^{13}C relaxation dispersion profiles. **C)** Secondary structure and stable isotope labeling pattern of RNA **2b**. Red data points 600 (150) MHz ^{1}H (^{13}C) frequency, blue data points 700 (176) MHz ^{1}H (^{13}C) frequency. Global fits are shown as a full-drawn line in the respective color.

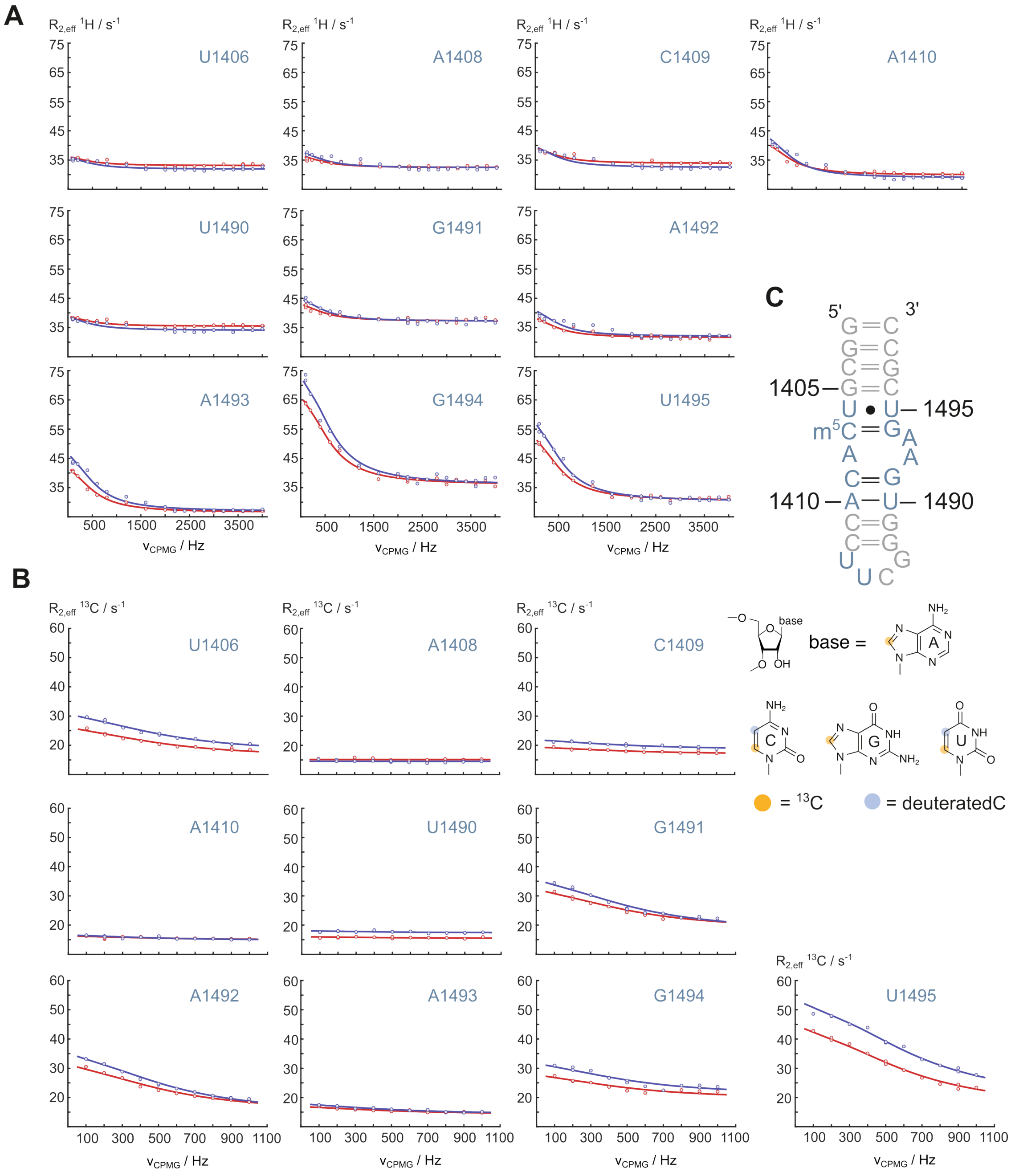

Supporting Figure 7. Assignment of imino proton and methyl group resonances of RNA **1b**. ^{15}N(1)-labeled guanosine and ^{15}N(3)-labeled uridine were site selectively incorporated to obtain start points for the assignment procedure. **A)** ^{1}H Imino spectrum of **1b** with assignments. **B)** ^{1}H-^{15}N-HSQC of selectively labeled **1b**. **C)** ^{1}H-^{1}H-NOESY spectrum of **1b**. D) Methyl HSQC of **1b** with assignment. **E)** Secondary structure and stable isotope labeling pattern of RNA **1b**. All spectra were acquired at 10°C.

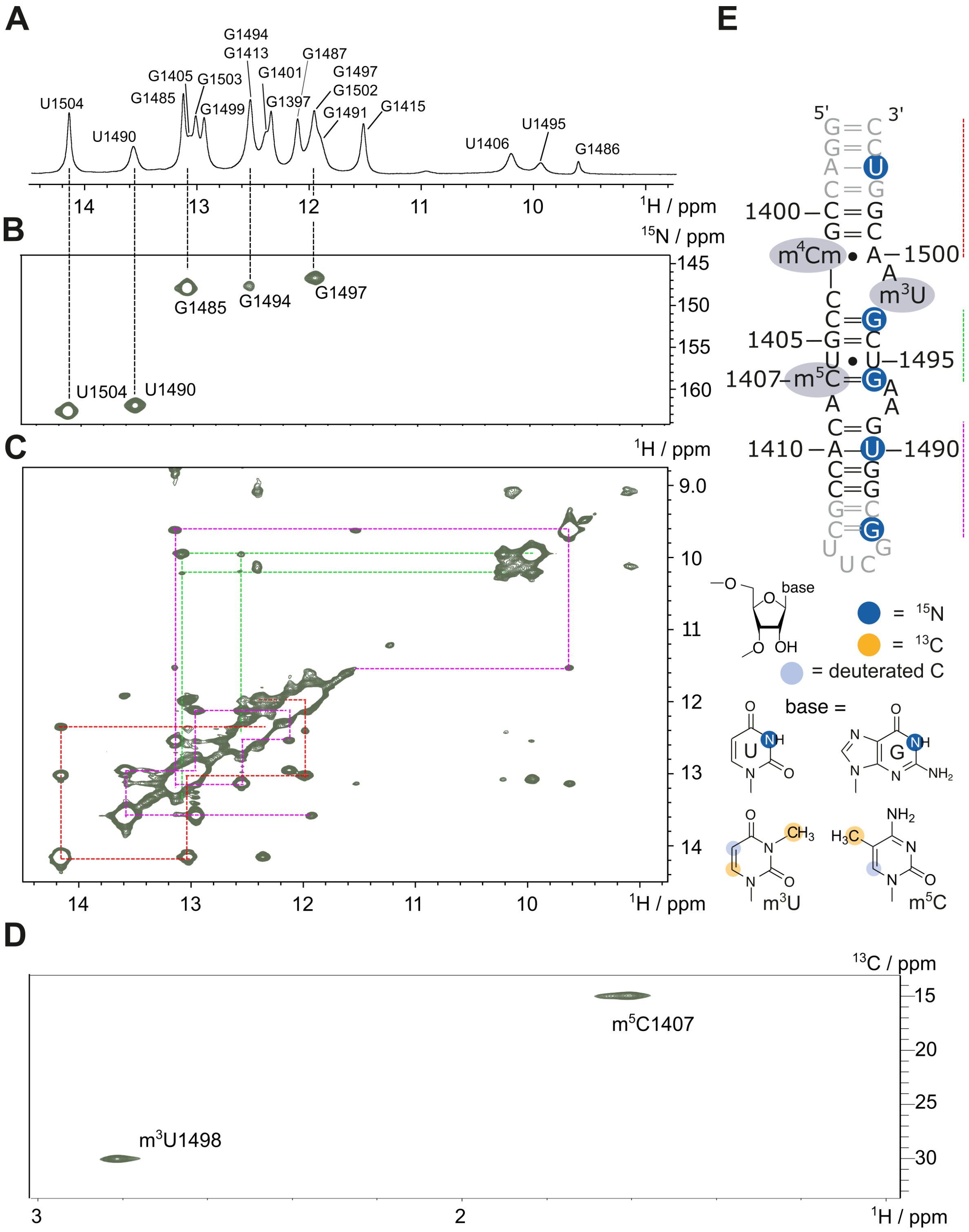

Supporting Figure 8. Stabilization of ES and GS by m⁵C determined via UV melting curves analysis. **A)** Wildtype A-site with and without m⁵C modification and the respective melting curve analysis to give the thermodynamic parameters. **B)** Comparison of wt A-site C1407 m⁵C1407 melting points. **C)** Excited state mimic with and without m⁵C modification and the respective melting curve analysis to give the thermodynamic parameters. **D)** Comparison of ES mimic C1407 m⁵C1407 melting points.

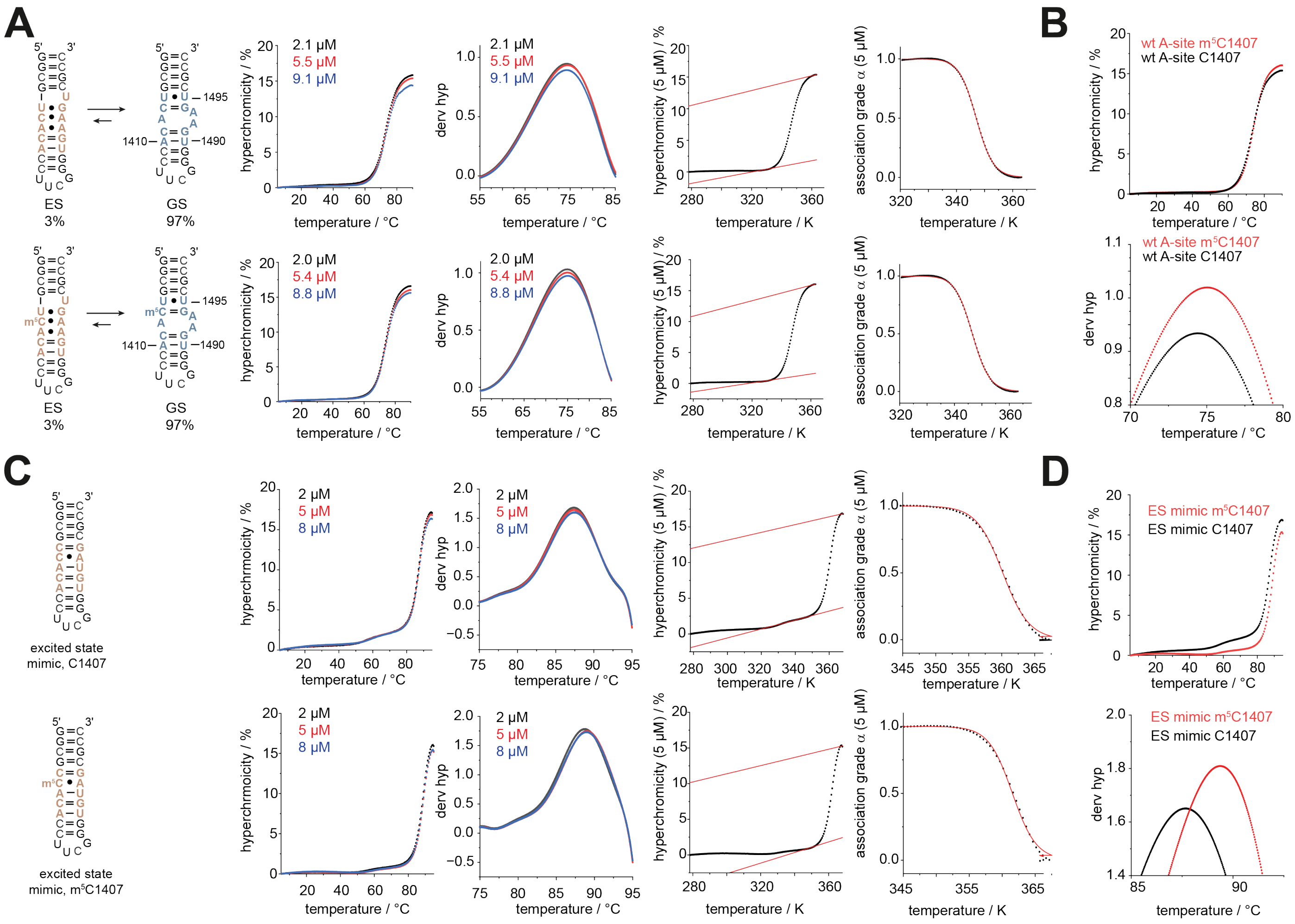

Supporting Figure 9. Influence of m^5C1407 on the folding landscape of the A-site RNA. **A)** Exchange process of the A-site RNA and the kinetic and thermodynamic parameters. **B)** Schematic of thermodynamics and kinetic energy barriers of the refolding process in the presence and absence of the C5 methylation of C1407.

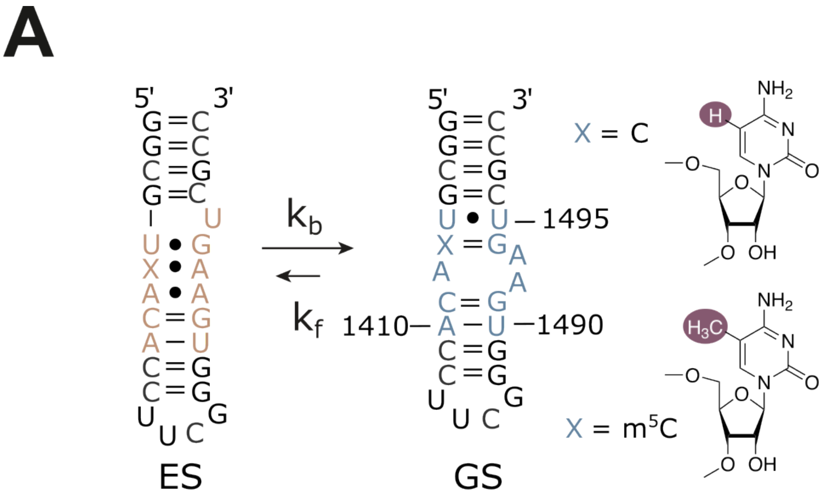

	k_{ex} / s^{1}	p_{ES} / %	τ_{GS} / ms
X = C	5492±241	3.2±0.7	5.7±0.6
X = m^5C	3480±139	3.1±0.2	9.3±0.9

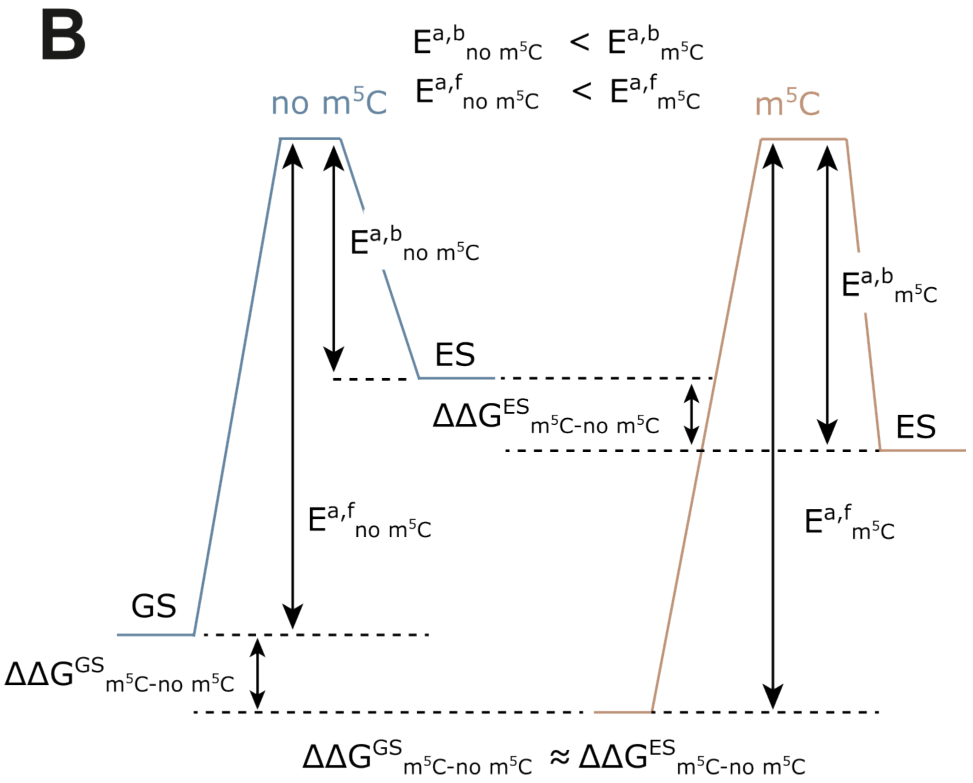

REFERENCES

(1) Delaglio, F.; Grzesiek, S.; Vuister, G. W.; Zhu, G.; Pfeifer, J.; Bax, A. NMRPipe: A multidimensional spectral processing system based on UNIX pipes. *Journal of Biomolecular NMR* **1995**, *6* (3), 277-293. DOI: 10.1007/BF00197809.
(2) Carver, J. P.; Richards, R. E. A general two-site solution for the chemical exchange produced dependence of T2 upon the carr-Purcell pulse separation. *Journal of Magnetic Resonance (1969)* **1972**, *6* (1), 89-105. DOI: https://doi.org/10.1016/0022-2364(72)90090-X.
(3) Bieri, M.; Gooley, P. R. Automated NMR relaxation dispersion data analysis using NESSY. *BMC Bioinformatics* **2011**, *12* (1), 421. DOI: 10.1186/1471-2105-12-421.
(4) d'Auvergne, E. J.; Gooley, P. R. The use of model selection in the model-free analysis of protein dynamics. *Journal of Biomolecular NMR* **2003**, *25* (1), 25-39. DOI: 10.1023/A:1021902006114.
(5) Marky, L. A.; Breslauer, K. J. Calculating thermodynamic data for transitions of any molecularity from equilibrium melting curves. *Biopolymers* **1987**, *26* (9), 1601-1620. DOI: https://doi.org/10.1002/bip.360260911 (acccessed 2025/04/14).
